# Persistent priming of hypothalamic microglia is associated with sensitization of the hypothalamic-pituitary-adrenal axis to acute stress, hyperactivity and behavioral response disruption in male rats

**DOI:** 10.3389/fimmu.2026.1828445

**Published:** 2026-06-30

**Authors:** Ana León-Rodríguez, María del Mar Fernández-Arjona, Carmen Pedraza, Manuel F. López-Aranda, Rick Visser, María D. López-Ávalos

**Affiliations:** 1 Departamento de Biología Celular, Genética y Fisiología, Facultad de Ciencias, Universidad de Málaga, Málaga, Spain; 2 Instituto de Investigación Biomédica de Málaga-IBIMA Plataforma Bionand, Málaga, Spain; 3 Departamento de Psicobiología y Metodología de las Ciencias del Comportamiento, Facultad de Psicología y Logopedia, Universidad de Málaga, Málaga, Spain

**Keywords:** acute stress, HPA axis, hyperactivity, hypothalamus, microglia, neuraminidase, neuroinflammation, priming

## Abstract

**Background:**

Central and peripheral inflammation are under intense investigation because of their increasing relationship with neuropsychiatric disorders and neurodegeneration. Microglia, the main component of the innate immune system within the brain, coordinate neuroinflammatory processes, so they are a major focus of attention. Recent research has highlighted the capacity of microglia to acquire immunological memory from previous inflammatory events, similarly to macrophages. Thus, primed microglia are sensitized cells capable of developing exacerbated inflammatory responses with potential deleterious outcomes.

**Objective:**

This study investigated the long-term consequences of a single acute neuroinflammatory event on hypothalamic sensitization of the stress response, underscoring microglial involvement.

**Methods:**

Neuroinflammation was provoked in rats by a single intracerebroventricular injection of microbial neuraminidase (NA). Three months later, they were exposed to acute stress consisting of forced swimming. Hypothalamic inflammatory activation and hypothalamic-pituitary-adrenal (HPA) axis response were assessed, along with rats´ behavior.

**Results:**

Acute stress provoked a heightened corticosterone response in rats that had undergone neuroinflammation compared to saline-injected rats, indicating HPA axis sensitization. In the hypothalamus of NA-injected rats examined 12 and 48 hours after stress, gene expression of neuropeptides CRH and AVP was decreased, while glucocorticoid receptor expression was increased. CRH protein stores also increased, as did enzymes involved in endocannabinoid synthesis. These results suggest molecular signs of altered HPA-axis feedback regulation. In parallel, gene expression of inflammation-related genes revealed a moderate enhancement of inflammation after acute stress, which was more evident in the amygdala than in hypothalamus and was still detected 48 hours after stress. Moreover, comprehensive morphological analysis of microglia located in paraventricular nucleus and basolateral amygdala revealed a more reactive microglial profile, consistent with priming. Open field evaluation revealed disorganized behavior characterized by hyperactivity, disinhibition, increased arousal, stress reactivity, and impaired risk assessment.

**Conclusion:**

A past acute neuroinflammatory event sensitizes the HPA axis, leading to augmented corticosterone response, molecular signs of altered HPA axis feedback regulation, and a complex behavioral response characterized by hyperactivity and increased arousal, all of which depict maladaptive stress reactivity. Long-lasting priming of microglial cells located in the hypothalamus, displaying an enhanced activation upon stress exposure, could contribute to those alterations.

## 1 Introduction

During the last decades it has become clear that brain inflammation is tightly associated with neurological diseases and neurodegeneration. In the nervous system inflammation is mostly coordinated by microglial cells, with the participation of other glial cells (particularly astrocytes) and peripheral immune cells that may be recruited as well (1, 2). Microglial cells, which actively survey the neural tissue milieu, become activated upon detection of a wide variety of molecular cues that indicate injury, infection or cell damage within the brain (3–5). Moreover, peripheral immune activation is conveyed to the brain, triggering microglial reaction as well (6). Inflammation is an adaptive innate immune response aimed at protecting tissues from damage or foreign agents. Therefore, once it is initiated, it peaks during the first days and progressively declines until tissue homeostasis is restored, which may take a few days or weeks depending on the nature and intensity of the initial stimulus. Cytokines, chemokines and prostaglandins are key mediators released by microglial cells in this process. Besides the restoration of tissue homeostasis, successful neuroinflammation includes the proper return to basal levels of all these mediators, and the regain of the surveilling profile by microglial cells. In fact, chronic or dysregulated neuroinflammation has been associated with a variety of neurological disorders and neurodegeneration (7–10).

Furthermore, it has recently been demonstrated that, similar to the immune memory developed by cells involved in the adaptive immune response, microglia may acquire a memory of past inflammatory events (11). Immune memory of microglia/macrophages manifests in two fashions: priming or sensitization, where the response to a second challenge is increased, and desensitization or tolerance, where the response to a subsequent insult is diminished (11). Persistent epigenetic modifications seem to underlie both functional states which, in the case of primed microglial cells, influence the development of neurological diseases (12, 13). A single severe neuroinflammation event, either of central or peripheral origin, is sufficient to prime microglial cells to future stressful or immunological challenges, therefore representing a risk factor for neurodegeneration, neurological diseases and mood disorders (10, 13–16).

Besides the well-established role of microglia in neuroinflammation, new relevant functions are being unveiled. Microglia are involved in neurogenesis and remyelination, acting as efficient phagocytes to clear cells or debris. Moreover, they are able to sense and modify neuronal activity, and are crucial in remodeling synaptic contacts and neuronal circuits (17–21). Therefore, microglia reactivity or dysregulation can affect the normal functioning of neural circuits.

Neuroinflammation of central or peripheral origin has been associated with psychiatric and mood disorders. Inflammation and disrupted neural functions seem to provide positive feedback each other in a pernicious loop, making it challenging to discern if one of them was first and gave rise to the other (reviewed, among others, in 22–27). In fact, a fraction of patients with major depressive disorder that do not respond to classic antidepressants show abnormally high levels of cytokines (28). Similarly, a subgroup of people with schizophrenia present increased peripheral cytokines (29). The neurological consequences that the recent COVID-19 pandemic imposed on a portion of patients represents another example (30, 31). Multiple studies have been able to pinpoint microglia as major players (32–35), with the potential contribution of peripheral immune cells that may infiltrate the brain (1, 2, 36, 37), thus identifying promising targets for novel treatments (9, 38–41).

Glucocorticoids are essential hormones with pleiotropic functions in virtually all types of tissues, whose major role is to deploy all animals´ resources to cope with stressful situations. In response to physiological or psychological stress, transient elevations of glucocorticoids can initially enhance immune responses, mobilize energy stores and augment cognitive resources to successfully face the stressful situation. Afterward, a proper return to basal glucocorticoid levels is required to prevent organism attrition and allostatic load due to the sustained effects of these hormones. Shortly after stress, moderate physiologic levels of glucocorticoids have been shown to enhance the immune response, which facilitates adaptation to the stressor (42, 43). However, sustained elevated levels are effective suppressors of the immune system, which has led to the use of glucocorticoids as anti-inflammatory and immunosuppressive agents. Also, they suppress their own production, acting as negative feedback regulators of the hypothalamic-pituitary-adrenal (HPA) axis, thus keeping a basal glucocorticoid tone that is relevant for multiple physiologic functions (44–46).

Altered levels of glucocorticoids have been extensively described in mood and psychiatric disorders. Interestingly, patients with Cushing’s syndrome, an endocrine disorder characterized by hypercortisolism, present psychiatric symptoms and cognitive deficits (47). Elevated glucocorticoids are also a common feature in major depression and schizophrenia, and have been related to the hippocampal atrophy, what could explain concurrent memory deficits (48–50). On the contrary, post-traumatic stress disorder (PTSD) patients also present memory impairments, however with normal or even low cortisol levels (51). These facts evidence that neurological alterations associated to glucocorticoids may arise from different mechanisms. For example, glucocorticoids as well as chronic stress inhibit hippocampal neurogenesis, what has been related to memory impartment, behavioral alterations (52, 53) as well as to anxiety and depression (54–57). The functionality of glucocorticoid receptors, which are highly expressed in various brain regions (such as hippocampus, amygdala, hypothalamus and prefrontal cortex) is frequently impaired in these pathologies, what may lead to glucocorticoid resistance and hyperactivity of the HPA axis (58–61). Finally, early exposure (pre- or postnatal) to high levels of glucocorticoids has been proposed to underlie the pathogenesis of schizophrenia, which is considered a neurodevelopmental disease (50, 62, 63). Therefore, dysfunction of the HPA axis is a widely recognized feature of psychiatric and mood disorders.

All this evidence have fueled investigations aimed to find out a relationship between inflammation, HPA axis activation and neuropsychiatric disorders like depression, anxiety, PTSD, autism or schizophrenia. Early studies showed the capacity of cytokine interleukin-1 (IL-1) to induce HPA axis activation in rodents, and the influence of glucocorticoids in such effect, demonstrating an interactive circuit between immune mediators and neuroendocrine axis (49, 64–66). From then on, evidence has accumulated pointing that immune activation is tightly linked to both the neuroendocrine systems that govern the stress response at the periphery, and also to brain structures that dictate conservative behaviors (6, 23, 66). Understanding these intricate interactions should shed light to unveil the pathophysiology of neurological and psychiatric disorders. In this scenario, brain resident microglia have emerged as major players (reviewed in 32–35, among others, 67). Microglia rapidly reacts upon peripheral or central infections, traumatic brain injury (TBI), stroke or acute psychologic stressors, what has been shown in brain regions related to emotions, mood and cognition such as the hippocampus (68), the prefrontal cortex (69) and, to a lesser extent, hypothalamus (14, 41, 70–72) and amygdala (73–76).

As mentioned above, acute activation of microglia may lead to their transition to a primed status, which could compromise their response to further challenges. For instance, TBI primes microglial response to both immune and psychologic stressors, resulting in increased inflammation, depression and sickness long after injury (15, 77–79). Similarly, we have previously shown that acute neuroinflammation provoked by a single intracerebroventricular (ICV) injection of microbial neuraminidase (NA) induces microglial priming in the hypothalamus. Moreover, hypothalamic arcuate nucleus alterations and energy balance disturbances concur with microglial priming, changes that remarkably linger for at least three months (14, 41, 71). Because the hypothalamus is at the core of major neuroendocrine axis, we hypothesize that priming of hypothalamic microglia may jeopardize endocrine functions at the periphery. In fact, while the consequences of TBI have been mostly focused on cortical injuries, evidence points to persistent HPA axis disturbances, possibly as a result of widespread damage due to secondary injury and neuroinflammation (77, 80–83). Besides, endocrine disorders and hypopituitarism have been reported in TBI patients long after injury (77, 84–87). Using the model of acute neuroinflammation provoked by microbial NA, here we focused on the long-term consequences of hypothalamic microglial priming on the HPA axis response to acute psychologic stress. Endocrine and behavioral alterations, along with HPA axis dysregulation, are reported, indicating that previous neuroinflammation and microglial priming represent risk factors for the outcome of future stressful challenges.

## 2 Materials and methods

### 2.1 Animals

Adult (≈ 3 months) male Wistar rats were acquired from Charles River Laboratories (France). Animals were housed in groups of three under standard conditions: 12-hour light/dark cycle, 23 °C and 60% relative humidity, and were allowed access to food and water *ad libitum*. All experimental procedures complied with Spanish legislation (RD 53/2013 and RD 118/2021) and European Union regulations (Directive 2010/63/EU). Ethical approval was received from the Ethics Committee of University of Málaga and the regional authorities (Junta de Andalucía, Ref. 04/10/2018/145). Procedures and animal handling were planned to minimize both the number and the suffering of animals used in the experiments.

### 2.2 Experimental design

**Figure 1:**
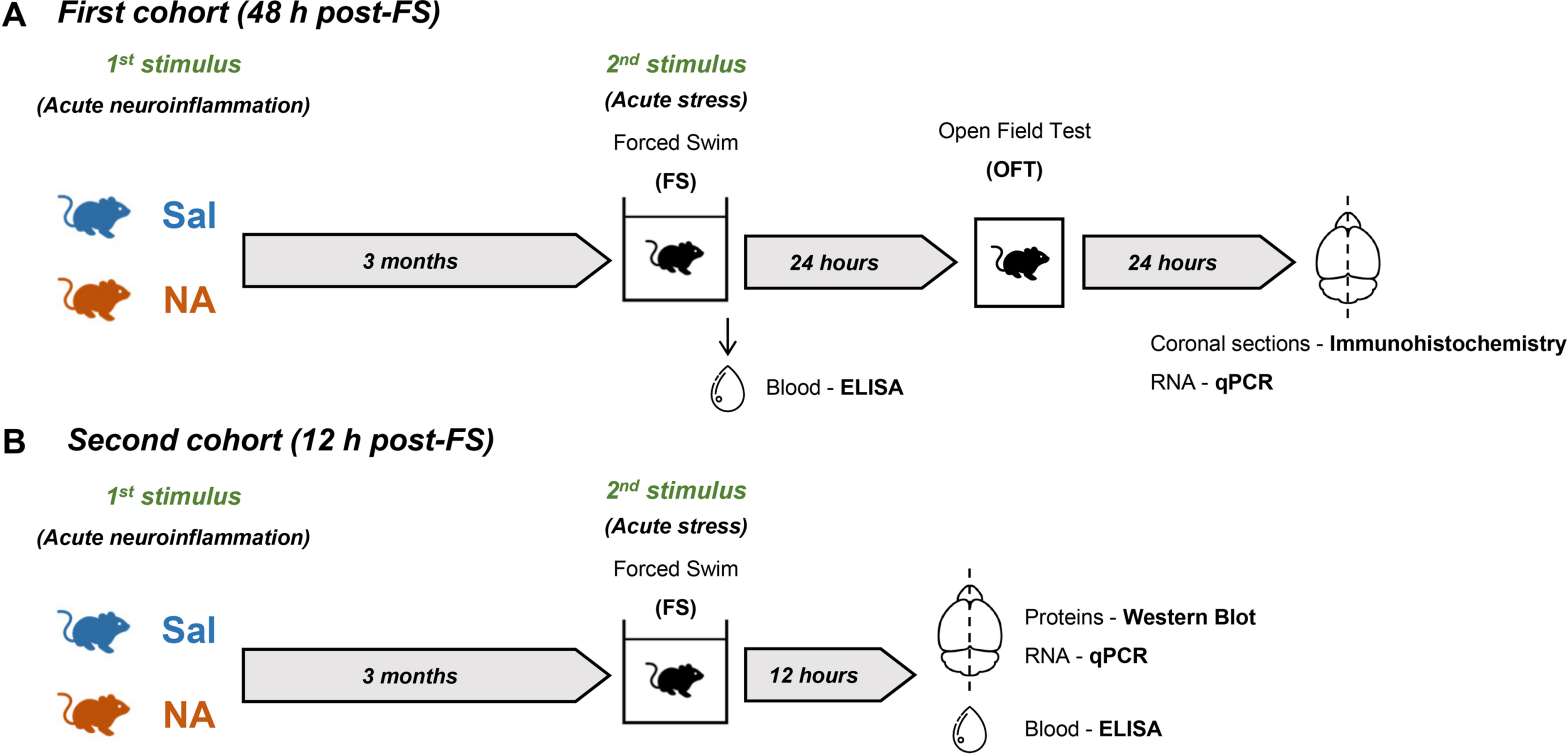
Scheme of the experimental design. Male rats were intracerebroventricularly (ICV) injected with neuraminidase (NA) to induce acute neuroinflammation; control rats were injected with saline (Sal). Three months later, all rats were exposed to a 6-minute forced swimming (FS) as an acute stressor. **(A)** In the first cohort of rats, blood samples were collected 10- and 30-minutes post-FS to assess fast HPA axis activation by measuring plasma corticosterone. Twenty-four hours after FS the animals performed an open field test (OFT) for behavioral assessment, and they were euthanized 24 h later. The brains were bisected along the midline and each hemisphere was used to analyze both the inflammatory and the HPA axis status either by immunohistochemistry or qPCR. **(B)** In the second cohort rats were euthanized 12 hours after FS. Blood samples were taken at the moment of sacrifice; the brains were bisected along the midline and each side used for studying the inflammatory and the HPA axis status 12 hours post-FS, either by western blot or qPCR.

The animals underwent an experimental design consisting of two neuroinflammatory challenges applied three months apart (**Figure 1**). The first one was the intracerebroventricular (ICV) injection of neuraminidase (NA), or saline in the case of control rats. Rats were allowed to recover for three months and then exposed to an acute stress event of forced swimming (FS).

Two cohorts of rats were used, depending on the schedule of sampling/procedures after the FS episode. For the first group (**Figure 1A**; n = 19 rats: 10 saline-injected; 9 NA-injected) blood samples were collected 10- and 30-minutes post-FS. Behavioral assessment was performed 24 hours later using the open field test (OFT). Animals were euthanized the next day (48 hours after FS). The brains were obtained and sectioned along the sagittal plane, with one hemisphere designated for histological studies and the other for qPCR. It is worth mentioning that unfortunately blood samples collected at the moment of euthanasia were lost.

Because brain tissue collection in the previous experiment was done at 48 h post-FS, the second cohort (**Figure 1B**; n = 12 rats: 7 saline-injected; 5 NA-injected) was intended to investigate protein/gene expression at a time point closer to the acute stress. Thus, euthanasia was conducted 12 hours post-FS and blood samples were collected at that moment. Similarly, the brains were divided in the sagittal plane and either side devoted to western blot and qPCR analyses.

### 2.3 Intracerebroventricular injection

Rats received an ICV injection of neuraminidase (NA) to induce acute neuroinflammation, as previously described (88). They were anesthetized with a solution of ketamine/xylazine (80 and 12 mg/kg respectively; Sigma Aldrich) and placed in a stereotaxic frame. A single dose of NA from *Clostridium perfringens* (Sigma-Aldrich, N3001) dissolved in sterile 0.9% saline solution (500 mU in 20 μL) was administered in the right lateral ventricle. The infusion was performed using a pump at a rate of 2 μL/minute over a 10-minute period. Control animals received an equivalent volume of sterile saline solution. Animals were monitored during recovery from anesthesia for signs of pain or distress. A full description of this method is available in **Supplementary Methods**.

### 2.4 Forced swimming and blood sampling

All rats underwent a 6-minute FS session, used here as an acute psychological stressor. Standard transparent plastic swim cylinders (50 cm height and 25 cm diameter) were used, filled with water to a depth sufficient to prevent the animals from escaping while maintaining a vertical swimming position. The water temperature was maintained at 22–25 °C to minimize thermal stress. Afterwards, rats were removed from water and thoroughly dried with towels to avoid hypothermia.

To assess the activation of the HPA axis after the acute stress, blood samples were retrieved from the tail vein for corticosterone (CORT) determination (only in the first cohort of rats). Two samples were obtained from each rat, at 10 and 30 minutes after FS. Finally, animals were returned to the original housing conditions.

### 2.5 Open field test

The open field test (OFT) was employed as a multidimensional behavioral assay to evaluate locomotor activity, exploratory behavior, emotional reactivity, and anxiety-related ethological responses, rather than as an isolated or definitive measure of anxiety. This test was conducted 24 hours after exposing the rats to the acute stressor. The arena consisted of a square box (90 × 90 cm surface area, 45 cm high walls) made of gray plastic. Each animal was placed at the center of the arena, and allowed to move freely for 5 minutes. A video camera positioned 2 meters above the box recorded the 5-minute sessions. Fecal boli were collected at the end of each 5-minute session, and the arena was cleaned with 70% ethanol before the next trial to prevent olfactory cues.

Using a video tracking system (Ethovision XT 7, Noldus, Wageningen, Netherlands), the following spatiotemporal parameters were analyzed in the recorded OFT sessions: (1) total distance traveled (cm), (2) locomotion speed (cm/s), (3) time spent in the center of the arena (s), (4) time spent in the periphery (s) and (5) immobility time. The time-to-frequency ratio in the center was also calculated.

Additionally, ethological behaviors during the OFT were manually scored by a blinded observer using the Raton Time 1.0 software (Fixma S.L., Valencia, Spain). The assessed behaviors in the rats included: (1) rearing and (2) rearing with support, which refer to the time spent exploring the environment while standing on its hind legs without support (rearing) or supporting itself against the walls of the arena (rearing with support); (3) freezing, or periods of immobility when only respiratory muscles move; and (4) grooming, which describes self-directed, sequentially patterned hygiene behavior.

For each behavior, the following metrics were calculated: duration, frequency (number of episodes within 5 minutes), mean time (or time-to-frequency ratio) and latency (time spent from placement in the arena to the first episode of the behavior lasting more than 2 seconds).

### 2.6 Sacrifice and tissue sampling

Rats were deeply anesthetized (solution of ketamine/xylazine, 80 and 12 mg/kg respectively; Sigma Aldrich) and blood samples were collected from the heart using a syringe. Then, rats were transcardially perfused with cold saline containing 10 IU/mL of heparin to clear the blood from tissues. Afterwards, the brain was removed and divided along the midline. The right hemisphere, designated for histological studies, was immersed in 4% paraformaldehyde at 4 °C overnight. The left hemisphere, intended for either RNA or protein extraction, was snap-frozen in dry ice and stored at −80 °C.

The brain regions of interest for RNA and protein extraction were microdissected using a rat brain matrix (RWD, 800-00147-00) and a 1 mm tissue biopsy punch. The microdissected samples obtained were 1) placed in RNAse-free tubes and processed for RNA isolation, or 2) placed in low protein binding tubes and processed for protein extraction. The dissected samples were stored at -80 °C until further processing. A full description of this method is available in **Supplementary Methods**.

### 2.7 Immunohistochemistry

The brains destined to histological studies were sectioned using a vibratome (Leica VT1000S) to obtain 40-μm coronal sections, which were organized into serial collections and stored in PBS. Sections containing the basolateral amygdala (BLA) and the paraventricular nucleus (PVN) of the hypothalamus were selected for immunohistochemistry (between − 1.5 mm and − 2.0 mm from Bregma approximately).

For IBA1 and GFAP immunostaining free floating sections were washed with PBS and incubated in PBT solution (0.3% bovine serum albumin and 0.3% Triton X-100 in PBS pH 7.3) to block the non-specific binding sites. Primary antibodies (rabbit polyclonal anti-IBA1 1:500, Wako 019-19741; rabbit polyclonal anti-GFAP 1:10000, Dako G9269) were incubated at 4 °C overnight, followed by secondary biotinylated antibody (goat anti-rabbit 1:1000, Pierce) and ExtrAvidin**^®^**-Peroxidase (1:1000, Sigma-Aldrich E2886). Peroxidase was visualized incubating the sections during 10 minutes with 0.05% diaminobenzidine (DAB) and 0.03% hydrogen peroxide in PBS.

As negative control for immunohistochemistry, primary antibodies were omitted. A full description of this method is available in **Supplementary Methods**.

### 2.8 Immunofluorescence

For CRH and IBA1 double fluorescent immunostaining, the primary antibodies used were rabbit polyclonal anti-CRH (1:5000, BMA Biomedicals T-4037) and goat polyclonal anti-IBA1 (1:700, Abcam ab5076). As secondary antibodies, donkey anti-rabbit Alexa 488 and donkey anti-goat Alexa 568 (1:1000, Thermo-Fisher A21206 and A11057, respectively) were employed. A 20-minutes wash with PBS containing DAPI (0.2 µg/ml, Sigma-Aldrich D9542) was employed to stain cellular nuclei. The sections were washed with PBS, mounted onto gelatin-coated slides and coverslipped using the anti-fading agent Mowiol 4-88 (Calbiochem/EMD Chemicals). As a negative control for immunohistochemistry, primary antibodies were omitted.

### 2.9 Image acquisition

For cell counts, images of IBA1 and GFAP stained sections were acquired using an Olympus VS120 scanner microscope with a UPLSAPO 20× objective (pixel side: 0.2857 µm; TIFF format).

For the morphometric analysis of microglia, high-resolution images were obtained using a UPLSAPO 60× oil immersion objective (pixel side: 0.015 µm; TIFF format). For each microscopic field a series of 20 images, each 1 μm thick, was captured in multi-plane virtual-Z mode, covering a total depth of 20 μm in the tissue section. The images were later aligned and combined, rendering a highly detailed image of the cells and their branches.

CRH and IBA1 double immunofluorescence images were acquired using a 40× objective on an inverted confocal microscope (LEICA SP5 II). Fluorescence images were captured with 11–15 z-stack planes, spaced 2 µm apart. Two sections per animal were used to quantify CRH+ labeling. To ensure consistency across images, sub-volumes of six z-stack planes were selected. Image quantification was performed using a macro in the FIJI software, measuring the integrated density for each z-plane and calculating the mean integrated density for each image. Two distinct CRH staining patterns were identified: one corresponding to the soma of CRH+ cells and another presenting a punctate-like distribution. For this study, only the somatic CRH staining was analyzed and presented.

### 2.10 Cell counts

The total number of cells in each delimited area of interest (PVN and BLA) was obtained manually using the software plugin Cell Counter for FIJI. The coronal sections used ranged between -1.44 and -1.92 mm relative to Bregma. Two sections per animal were used for cell counting in each brain structure; the mean value of each animal was calculated and the results presented as number of cells/mm^2^.

### 2.11 Image processing and morphological analysis

Microglial cells in the same two regions (PVN and BLA) were evaluated morphologically using the high-resolution images. Individual cells were selected and cropped from TIFF images, according to the next criteria: (1) random selection, in the PVN starting at the wall of the third ventricle and moving towards the parenchyma, and in the BLA starting at the upper vertex and moving downwards; (2) exclusion of cells overlapping with neighboring cells; and (3) inclusion of cells with complete soma and branches. N = 10 cells were selected per animal from each region, resulting in a total of about n = 100 cells sampled per experimental group from both the PVN and the BLA.

The pictures with individual cells were processed for their morphological analysis using the free software FIJI. For each image, the following steps were followed: (1) split the image into red, blue and green channels; the blue channel, which heightens the brownish IBA1 staining, was selected and used from this point forward. (2) Enhance the cell profile. (3) Obtain a binary image using a pre-established threshold value in the grayscale, consistent across all images. (4) Edit manually the binary image to accurately represent the original color image, connecting the broken branches and removing debris from nearby cells. (5) Get three different images of the binary cell: a filled shape, an outlined shape, and a skeletonized shape, which were then used to obtain the morphological parameters.

Three methods were employed for the morphological evaluation of microglia: Fractal analysis, Skeleton analysis and Sholl analysis.

The Fractal analysis was carried out with the FracLac plugin for FIJI (Karperien A., version 2.5), utilizing the filled and outlined shapes. This analysis generated 15 parameters: cell area, cell perimeter, lacunarity, fractality, circularity, convex hull area, convex hull perimeter, convex hull circularity, density, roughness, span ratio, bounding circle diameter, maximum span across the convex hull, the ratio of maximum/minimum convex hull radii and mean radius. These parameters are further detailed in (89).

Skeleton analysis was made with the free plugin AnalyzeSkeleton for FIJI (Arganda-Carreras I., version 3.4.2) (90) using the skeletonized image. Nine parameters were yielded with this method: branches, junctions, endpoint voxels, junction voxels, slab voxels, average branch length, maximum branch length, triple points and quadruple points.

Finally, Sholl analysis was performed with the free plugin SNT for FIJI (Longair M., version 4.3.0-pre-release3) (91) using the skeletonized profile. This method is based on the intersections of the cell skeleton with a series of concentric circles drawn from the soma/centroid of the cell. Thus, the number of branch intersections and their distance from the soma/centroid were obtained, as well as other four parameters: critical radius, maximum number of intersections, primary branches and ramification index. Further detailed explanations of each method and the parameters obtained through Skeleton and Sholl analysis are available in Leon-Rodriguez, Grondona (41).

### 2.12 RNA isolation

RNA was isolated from brain microdissected tissue samples obtained from hypothalamus and amygdala using RNAzol (Molecular Research Center Inc., RN 190). 0.5 ml of the reagent was added to 50 mg of tissue, and mechanical disruption was performed using the pipette tip and a plastic micro pestle. The extraction was done following manufacturer’s instructions. The final RNA pellet was dissolved in 30 μl of RNAse-free water, and stored at -80 °C. RNA concentration was determined using a Nanodrop equipment.

### 2.13 Reverse transcription

RNA samples were diluted with RNAse-free water to homogenize concentration across all samples to 50 ng/μl. Each reverse transcription (RT) reaction tube contained 8 μl of RNA sample and 2 μl of PrimeScript RT Master Mix reaction kit (Takara, RR036A), thus resulting in a final reaction volume of 10 μl. The RT reaction was carried out for 15 minutes at 37 °C, followed by 5 minutes at 85 °C Finally, a 1:10 dilution was done with water, resulting a concentration of 4 ng of cDNA equivalents/μl. The cDNA samples were stored at -20 °C.

### 2.14 Quantitative PCR

The sequences of the genes of interest were found in the Genbank NCBI Reference Sequence and primers were designed using the Primer Blast program (**Supplementary Table 1**).

A real-time PCR technique based on SYBR Green I fluorescence dye was used to quantify the level of target mRNA. Reactions were carried out in a 96-well PCR plate. Each tube contained: 5 μl of the master mix (FastStart Essential DNA Green Master; Roche 06402712001), 1 μl of both primers (0.4 μM each), and 4 μl of sample cDNA (5 ng of cDNA/μl) in a final volume of 10 μl. The PCR reaction was performed using a LightCycler^®^ 96 equipment (Roche). Amplification curves, dissociation curves and quantification cycles (Cq) were retrieved from each target gene. Data were analyzed using the software provided with the LightCycler^®^ 96 equipment.

Before running the qPCR, the PCR efficiency (E) of each pair of primers was estimated by running amplifications with serial dilutions of the cDNA samples. E was obtained with the equation E = 10 ^[− 1/slope]^ – 1. The expression of each target gene was normalized to that of the reference gene glyceraldehyde 3-phosphate dehydrogenase (GAPDH), and was calculated with the following formula:


$$\text{Gene expression (relative to GAPDH)}={\left({\text{E}}_{target}\right)}^{\text{ΔCP}target}{/}^{\left({\text{E}}_{GAPDH}\right)}\text{ΔCP}GAPDH$$

Where E is the efficiency for each target gene, and ΔCP is the difference between the Cq values of the control and experimental sample, for both the target and reference genes.

### 2.15 Protein extraction

Microdissected hypothalamic tissue samples were homogenized with a tissue-lyser system (1.5 minutes at 30 Hzs^-1^) using 0.5 ml of ice-cold RIPA buffer [50 mM Tris-HCl, 150 mM NaCl, 1% Triton X-100, 0.25% sodium deoxycholate (NaDOC) and 1mM EDTA, pH 7.4] per 8 mg of tissue, combined with protease and phosphatase inhibitors [1 mM phenylmethylsulfonyl fluoride (PMSF), 1 μg/ml pepstatin, 5 μg/ml leupeptin, 5 μg/ml aprotinin, 10 μg/ml trypsin inhibitor, 1 mM sodium orthovanadate (NaOV_4_) and 1 mM sodium fluoride (NaF)]. After homogenization, samples were centrifuged at 12, 000 rpm for 15 minutes at 4 °C, and supernatants were collected. Protein concentration was determined by the Pierce™ bicinchoninic acid (BCA) protein assay (Thermo Fisher Scientific, 23225). For electrophoresis, 15 μl of the extract were mixed with 15 μl of gel loading buffer (125 mM Tris-HCl pH 6.8, 6% SDS, 30% glycerol, 160 mM DTT, and 0.01% bromophenol blue).

### 2.16 Western blot analysis

Protein extracts were loaded and separated in 4-12% polyacrylamide gradient gels (Bio-Rad Laboratories, 3450124), and then transferred to nitrocellulose membranes (Bio-Rad Laboratories, 1620115). The membranes were saturated to prevent non-specific binding. The following primary antibodies were incubated overnight at 4 °C: rabbit polyclonal anti-NAPE-PLD (1:1000, Abcam 95397); rabbit polyclonal anti-DAGLα (1:100, Biorbyt 156533); rabbit polyclonal anti-DAGLβ (1:100, Biorbyt 182976); rabbit polyclonal anti-NF-κB (1:500, Cell Signaling 8242S); rabbit polyclonal anti-NF-κB (Ser536) phosphorylated (1:1000, Cell Signaling 3033S); rabbit polyclonal anti-CRH (1:1000, BMA Biomedicals T-4037). As a reference protein, mouse polyclonal anti-γ-adaptin 1:2000 (BD Biosciences 610385) was used. Secondary antibodies were HRP-conjugated anti-rabbit or anti-mouse IgG (Promega, Madison, WI, USA). Protein bands were detected with Western Blotting Luminol Reagent kit (Santa Cruz Biotechnology, CA, USA) and quantified by densitometric analysis with the free software FIJI. The results were expressed as the ratio of target protein band to γ-adaptin band or, in the case of NF-κB, the ratio of phosphorylated NF-κB to total NF-κB.

A full description of this method is available in **Supplementary Methods**.

### 2.17 Corticosterone ELISA

Corticosterone levels were measured in plasma samples obtained from blood, using the commercial enzyme-linked immunosorbent assay (ELISA) kit DetectX^®^ Corticosterone (Arbor Assays™, K014-H1). The assay was run following the manufacturer’s instructions. Results were read using BioTek Epoch microplate spectrophotometer (Agilent Technologies, CA, USA) and processed with MyAssays online tool (also available with the ELISA Kit). The detection limit of the assay was 14.35 pg/ml (for high sensitivity test with 100 μl sample size).

### 2.18 Statistical analysis

Statistical analysis was performed using GraphPad Prism 9 software. Outlier data were identified with the ROUT method and removed from further analysis. To assess the validity of parametric methods to analyze our data sets, Kolmogorov–Smirnov (n < 50) and Shapiro Wilk (n > 50) normality tests and Levene homoscedasticity test were used. If the data met the assumption of normality and homoscedasticity, unpaired *t*-test was applied. Differences between groups were considered significant when *p*-value was < 0.05.

For data that did not meet the assumption of normality, a Kruskal–Wallis test for non-parametric data was employed. If the data met the assumption of normality but did not meet the assumption of homoscedasticity, unpaired *t*-test with Welch’s correction was used. In both situations, differences between groups were considered significant if *p*-value was < 0.05.

Histograms show the mean ± the standard deviation (SD), as well as the individual value points. For morphological parameters from microglial cells, data distribution is represented with violin plots, with an estimated density calculated by Kernel Density Estimation (KDE) method. The violin plots are truncated at the maximum and minimum values of each dataset and presented with medium smoothing.

Significant differences between groups are indicated with asterisks. *P*-values close to significance (*p*-value < 0.15) are also represented in the graphs.

## 3 Results

Our experimental design aimed to explore the potential impact of innate immune memory and microglial priming on the activation of hypothalamic-pituitary-adrenal (HPA) axis and behavior after an acute stress (**Figure 1**). It has been extensively characterized by our group that the ICV injection with neuraminidase (NA) provokes an acute neuroinflammatory process that peaks within the first few days and is largely solved after two weeks (88). However, mild behavioral alterations persist beyond this period (75). Furthermore, there is evidence of microglial priming remaining long after the ICV injection of NA, specifically in hypothalamus.

Primed microglia are usually not inflammatory and behave mostly as surveillant cells. Priming is attributed to epigenetic modifications not easily identifiable (12, 13). For this reason, well established markers of primed microglia are not available. Observing the microglia response after a second stimulation is widely considered an appropriate strategy to functionally identify priming. Thus, exposure to a novel inflammatory challenge (e.g. peripheral lipopolysaccharide (LPS), high fat diet or obesity) three months after the ICV injection resulted in enhanced microglial activation and neuroinflammation (14, 41, 71). This represents an experimental paradigm of two-hits or insults, with the singularity of permitting a long relapse time between both inflammatory insults. In the present work we used as a second insult an acute psychological stressor consisting of forced swimming during 6 minutes (FS). Immediately after (10 and 30 minutes post-FS) blood samples were drawn to assess HPA axis activation (plasma CORT measured by ELISA), and the next day behavior was evaluated in the open field. Rats were sacrificed the following day for brain collection, where the neuroinflammatory response was investigated by immunohistochemistry and qPCR (**Figure 1A**). Because with this experimental protocol most data about HPA-axis activation and neuroinflammation was obtained at 48 hours post-FS (relatively long after the application of the stressor), we repeated the same paradigm in a second cohort of rats that were sacrificed 12 hours after FS, using in this case the brains for protein (Western blot) and gene expression (qPCR) studies (**Figure 1B**).

### 3.1 Previous neuroinflammation enhances the activation of the HPA-axis after acute stress

**Figure 2:**
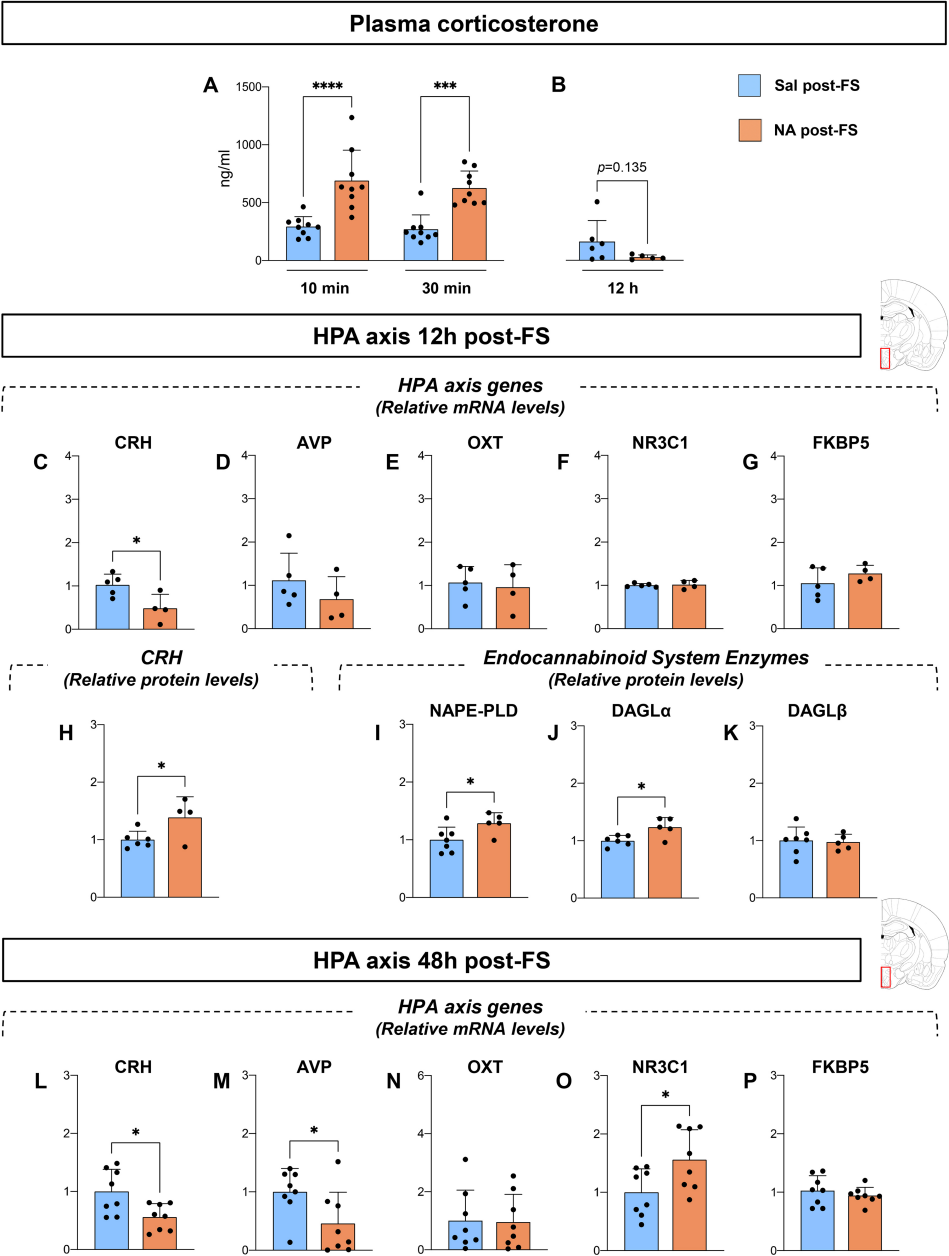
HPA axis activation due to NA-induced neuroinflammation following acute stress. Rats were neuraminidase (NA) or saline (Sal)-injected and 3 months later exposed to a 6-minute forced swimming (FS). Rats were euthanized either 12 or 48 hours after FS for tissue sampling. **(A, B)** Corticosterone levels in blood plasma taken at 10 and 30 minutes (from 1^st^ cohort) and 12 hours (from 2^nd^ cohort) post-FS. **(C–G, L–P)** The expression of HPA-axis related genes (neuropeptides CRH, AVP and OXT, glucocorticoids receptor NR3C1 and chaperone FKBP5) in the periventricular tissue of the hypothalamus was quantified by qPCR at 12 **(C–G)** and 48 **(L–P)** hours after FS; mRNA levels are shown relative to the expression of the housekeeping gene GAPDH. **(H–K)** Protein expression of CRH and key enzymes of the endocannabinoid system in the periventricular tissue of the hypothalamus collected 12 hours post-FS; protein levels were measured by western blot and expressed relative to γ-Adaptin. Histograms show the mean ± SD and the individual values of n = 4–6 animals (12 hours post-FS) and n=7–9 animals (48 hours post-FS) per group. *p < 0.05, ***p < 0.001, ****p < 0.0001. *p*-values close to significance (*p* < 0.15) are also shown.

To capture the peak levels of plasma CORT, blood samples were collected 10 and 30 minutes after FS. In ICV-saline injected control animals CORT levels 10 minutes post-FS reached 293.5 ± 86 ng/ml. Interestingly, CORT raised to 689.8 ± 263.5 ng/ml in those animals ICV-injected with NA 3 months before (**Figure 2A**), indicating a heightened HPA axis response. Similar results were obtained 30 minutes post-FS (270 ± 124.6 ng/ml in ICV-saline vs 627.3 ± 146.5 ng/ml in ICV-NA). In the cohort of rats sacrificed 12 h after FS, CORT levels in ICV-saline rats were 162.7 ± 74.3 ng/ml at the moment of sacrifice (12 h post-FS), indicating a progressive return to basal levels; NA injected rats showed a tendency to even lower CORT levels (27.45 ± 19.7 ng/ml), although the difference with control rats was not significant (**Figure 2B**). Unfortunately, blood samples collected at the moment of sacrifice of the first cohort (48 h post-FS) were lost, so data of CORT levels at this time point are not available.

We further examined other variables related to the mechanisms underlying HPA-axis activation and regulation, both 12 and 48 hours after FS; mRNA and protein levels were analyzed in periventricular hypothalamic tissue (**Figures 2C–P**). Twelve hours after FS those rats injected with NA 3 months earlier exhibited decreased mRNA levels of corticotropin releasing hormone CRH, a reduction that remained after 48 hours (**Figures 2C, L**). In parallel, CRH protein levels in NA injected rats were significantly increased (**Figure 2H**; **Supplementary Figure 1**), that suggests increased CRH accumulation (probably due to reduced secretion) in the hypothalamus. Similarly, arginine vasopressin (AVP) showed decreased levels of mRNA in NA-injected animals, although the difference with ICV-saline controls was significant only at 48 h post-FS (**Figures 2D, M**). On the contrary, the expression levels of the neurohormone oxytocin (OXT) were similar in both groups (**Figures 2E, N**). Regarding the glucocorticoid receptor gene NR3C1 and its chaperone FK506 binding protein 5 (FKBP5), both involved in the mechanism of CORT negative feedback, not significant differences were observed 12 h post-FS (**Figures 2F, G**); notably, NR3C1 expression was significantly increased in NA injected rats 48 h after acute stress (**Figure 2O**).

The endocannabinoid system is known to be one of the several mechanisms involved in the negative feedback regulation that glucocorticoids exert on the HPA axis, which is mediated by CORT membrane receptors (92). The two main recognized endocannabinoids are anandamide (AEA) and 2-arachidonoylglycerol (2-AG), whose main enzymes of synthesis are N-acylphosphatidylethanolamine-phospholipase D (NAPE-PLD) and diacilglycerol lipase (DAGL, which presents two isoforms DAGLα and DAGLβ), respectively (93). To have an insight into the negative regulation by CORT mediated by endocannabinoids in the HPA axis, we quantified the protein levels of the enzymes NAPE-PLD, DAGLα and DAGLβ by western blot (**Figures 2I–K**; **Supplementary Figure 1**). NAPE-PLD and DAGLα (**Figures 2I, J**), but not DAGLβ (**Figure 2K**), were slightly overexpressed in NA-injected and FS stressed rats compared to saline-injected controls, 12 h after applying the stress. These results point to an increased synthesis of AEA and 2-AG, and possibly an enhanced negative feedback of CORT mediated by these molecules, in rats previously injected with NA.

**Figure 3:**
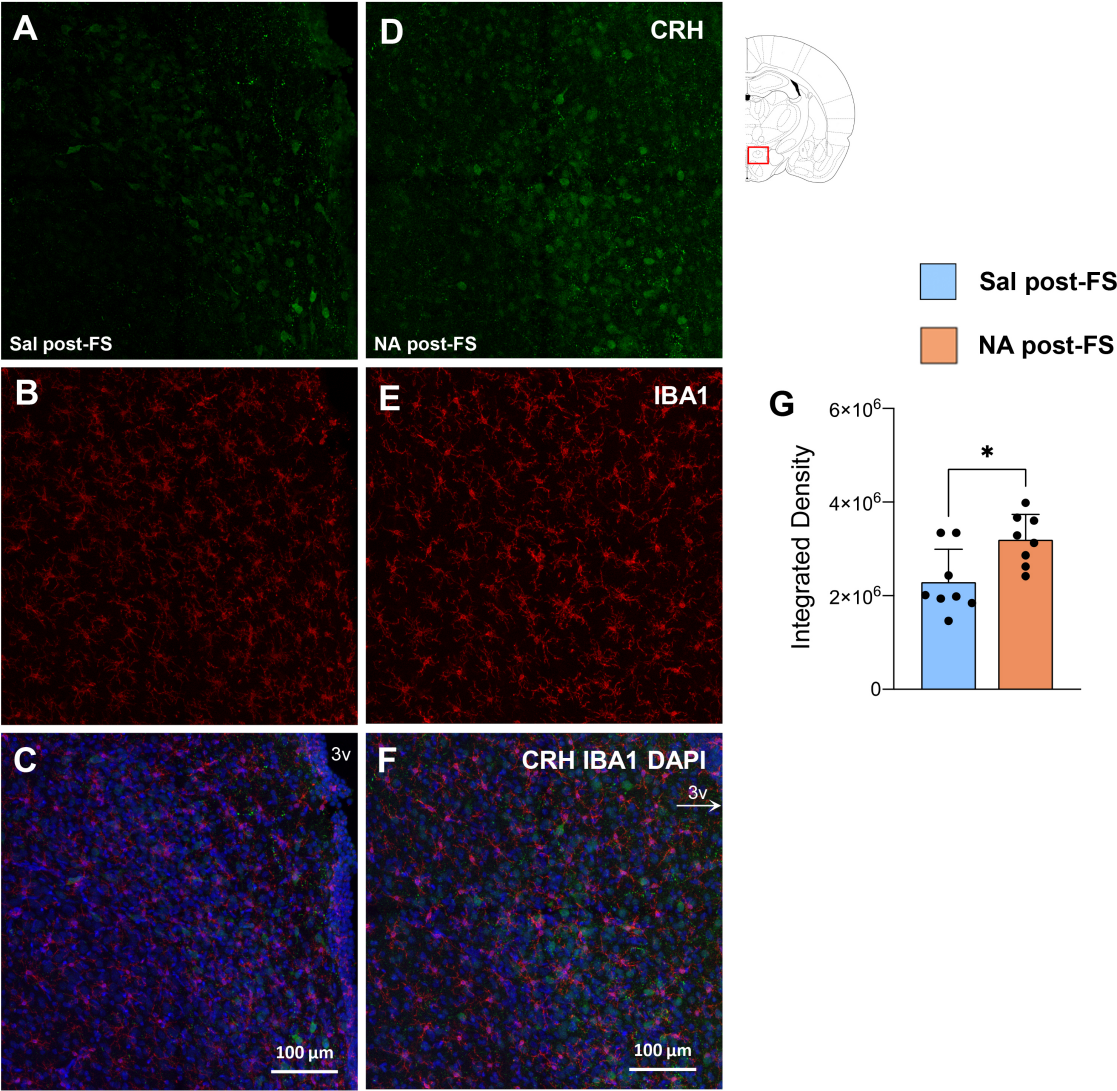
Immunohistochemistry for CRH in the hypothalamic PVN. Rats were injected with neuraminidase (NA) or saline (Sal) and exposed to a 6-minute forced swimming (FS) 3 months later. Rats were euthanized 48 hours after FS. Coronal brain sections at the level of the PVN were immunostained for CRH **(A–D)** and IBA1 **(B–E)**, and nuclei were stained with DAPI **(C–F)**. **(G)** Mean pixel intensity of the CRH label was quantified by image analysis of confocal images (containing 6 confocal z-sections of PVN). The histogram shows the mean ± SD and the individual values of n = 8 animals per group. 3v, Third ventricle. *p < 0.05.

CRH protein in the hypothalamic PVN was further assessed by immunofluorescence in the cohort of rats sacrificed 48 hours after FS (**Figure 3**). Quantification of the CRH label in the PVN revealed increased signal intensity in animals that received NA injection 3 months before (**Figures 3D–F**) compared to those injected with saline (**Figures 3A–C, G**), what is consistent with our previous western blot results (**Figure 2H**). Worth to mention is that immunostaining with IBA1 (an antibody that labels microglia and macrophages) evidenced an increased IBA1 label intensity in hypothalamic microglia of animals previously injected with NA (**Figure 3E**) compared to saline controls (**Figure 3B**), which is in accordance with an increased microglial activation. Changes is this cell population will be further explored in subsequent sections.

Overall, these results show an increased activation of the HPA axis (higher levels of CORT in plasma) shortly after FS in rats that suffered neuroinflammation three months before. Plasma CORT levels were normalized 12 h after FS in saline control rats (supposedly, but basal CORT levels prior to FS are not available), while NA-injected rats had CORT levels intriguingly (although not statistically significant) low. Also, these results provide evidence of enhanced feedback inhibition in the HPA axis of NA injected rats at 12 h post-FS (that is, reduced CRH and AVP gene expression and increased CRH protein accumulation, along with increased expression of endocannabinoids synthesis enzymes). Remarkably, several markers compatible with enhanced negative feedback regulation remained even 48 h after applying the acute stressor, as evidenced again by reduced CRH and AVP gene expression and increased CRH protein accumulation, as well as increased expression of the CORT receptor (NR3C1).

### 3.2 Neuroinflammation induced by acute stress is enhanced in rats that suffered NA-provoked neuroinflammation three months before

Previous studies from our group have highlighted increased neuroinflammation in animals that had undergone ICV injection of NA and its associated neuroinflammatory process months before (14, 71). Therefore, in order to study neuroinflammation in our two-hit paradigm with an acute FS stressor, we evaluated the expression of several neuroinflammation related genes. The areas selected for this study were the hypothalamic tissue adjacent to the third ventricle, where relevant hypothalamic nuclei (like PVN) with an important role in the stress response are located, and the amygdala, which is also crucial for the stress signaling and associated behavioral alterations.

**Figure 4:**
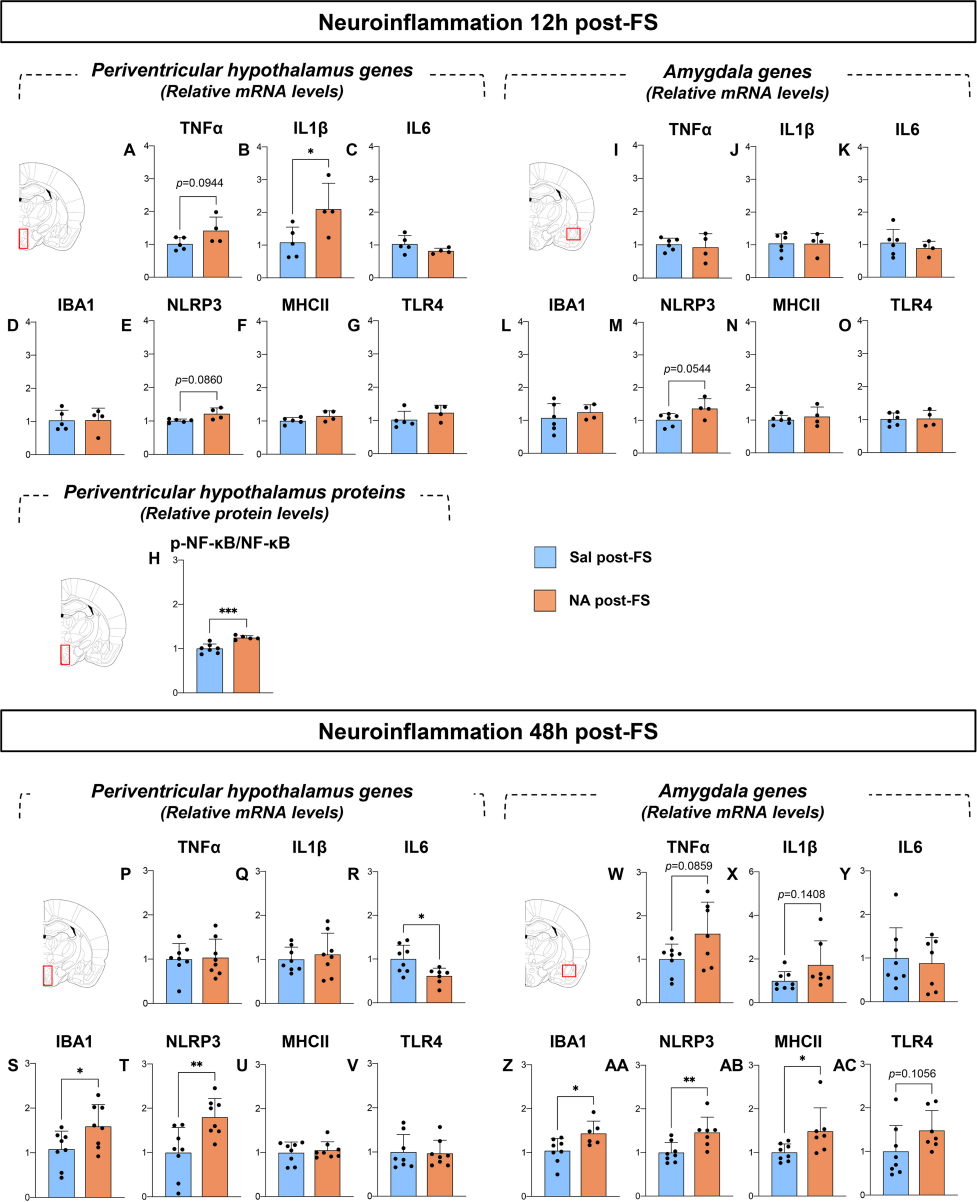
Assessment of neuroinflammation induced by acute stress in rats that suffered previous NA-injection. Rats injected with neuraminidase (NA) or saline (Sal) were exposed 3 months later to forced swimming (FS) and euthanized 12 **(A–O)** or 48 **(P–AC)** hours post-FS. Neuroinflammation was studied with qPCR in periventricular hypothalamus **(A–G; P–V)** and amygdala **(I–O; W–AC)**. mRNA levels of neuroinflammation-related genes were quantified by qPCR and represented relative to the housekeeping gene GAPDH. **(H)** Phosphorylated and non-phosphorylated NF-κB protein levels from the periventricular hypothalamus were measured by western blot; the ratio between them was calculated as an estimate of NF-κB pathway activation. The histograms show the mean ± SD and the individual values of n = 4–6 animals (12 hours post-FS) and n = 7–9 animals (48 hours post-FS) per group. *p < 0.05, **p < 0.01. *p*-values close to significance (*p* < 0.15) are also shown.

In periventricular hypothalamic tissue the expression of the cytokine IL1β was increased 12 h after FS in NA-injected rats (**Figure 4B**) compared to saline-injected controls, and tumor necrosis factor α (TNFα) and the inflammasome gene NOD-, LRR- and pyrin domain-containing protein 3 (NLRP3) showed the same trend, although not statistically endorsed (**Figures 4A, E**). In amygdala only NLRP3 showed the same trend (but not statistically significant; **Figure 4M**). Other genes analyzed (cytokine IL6, microglia/macrophage specific protein IBA1, major histocompatibility complex class II (MHCII), and toll-like receptor TLR4) were similarly expressed in NA-injected and saline-injected rats 12 h after FS. In the cohort of rats sacrificed 48 h after FS, IBA1 and NLRP3 expression were significantly increased in NA-injected rats, both in hypothalamus (**Figures 4S, T**) and amygdala (**Figures 4Z, AA**). Likewise, the expression of other genes was also increased (MHCII; **Figure 4AB**), or showed such trend (TNFα, IL1β, TLR4; **Figures 4W, X, AC**), but only in amygdala. Of note, the expression of the key proinflammatory cytokine IL6 remained largely unaffected; however, an intriguing reduction was observed in the hypothalamus of NA-injected rats 48 h (but not at 12 h) after applying the stressor.

To further study the neuroinflammatory profile, we evaluated the activation of the nuclear factor kappa-light-chain-enhancer of activated B cells (NF-κB) signaling pathway in periventricular hypothalamic tissue of rats in cohort 2, which were sacrificed 12 h post-FS. In this case the ratio of phosphorylated (activated) to non-phosphorylated (non-activated) NF-κB was quantified by western blot (**Figure 4H**; **Supplementary Figure 1**). Animals previously injected with NA exhibited a significantly increased ratio p-NF-κB/NF-κB, indicating increased pro-inflammatory signaling (**Figure 4H**).

Thus, our gene expression study revealed an enhanced inflammatory response to the acute stressor FS in those animals that suffered NA-induced neuroinflammation three months before, compared to the sham operated rats injected with saline. Even though the increased expression was not generalized for all genes assessed, in both brain regions analyzed (hypothalamus and amygdala) and at both times post-FS evaluated (12 h and 48 h), overall these data point to an enhanced inflammatory response, that can be detected even 48 h after applying the stressor. Interestingly, amygdala seems to be more prone to a delayed (48 h post-FS) inflammation provoked by this two-hit paradigm.

### 3.3 Microglial cells count and morphology point to increased microgliosis after acute stress in rats that suffered a previous NA-induced neuroinflammation

In former unpublished work (**Supplementary Figure 3**) we assessed the microglial reaction (in terms of number and morphology of the cells) in the neuroinflammation model based on the ICV injection of NA. This evaluation was carried out at a short time after the ICV injection (12 h), when neuroinflammation is in its acute phase, and long after (3 months), when the inflammatory parameters previously analyzed were indicating that neuroinflammation was largely solved (88). That study included both areas of interest in the present work, PVN and basolateral amygdala (**Supplementary Figure 3**). When compared to the ICV saline injected rats, shortly after NA-injection microglia underwent morphological changes in accordance with an increased activation state (**Supplementary Figure 3**; compare Sal 12h with NA 12h). Three months later most of those morphological changes had reverted (**Supplementary Figure 3**; compare Sal 3m with NA 3mo; and compare NA 12h with NA 3mo), but some mild differences remained (**Supplementary Figures 3D–H, K, O, P**); similar results were observed both in PVN and amygdala. These results suggested mild but long-lasting consequences of acute neuroinflammation on microglial cells, namely microglial immune priming, an issue that has been previously addressed by our team (75). Based on these previous works we hypothesized that the exposure to a second inflammatory insult (such as an acute psychological stressor) long after the initial priming event (three months later) could reactivate microglial cells in an exacerbated manner. Therefore, in the present work NA- and saline-injected rats were left undisturbed for three months before applying acute FS; their brains were collected 48 h later for histological studies (cohort 1).

**Figure 5:**
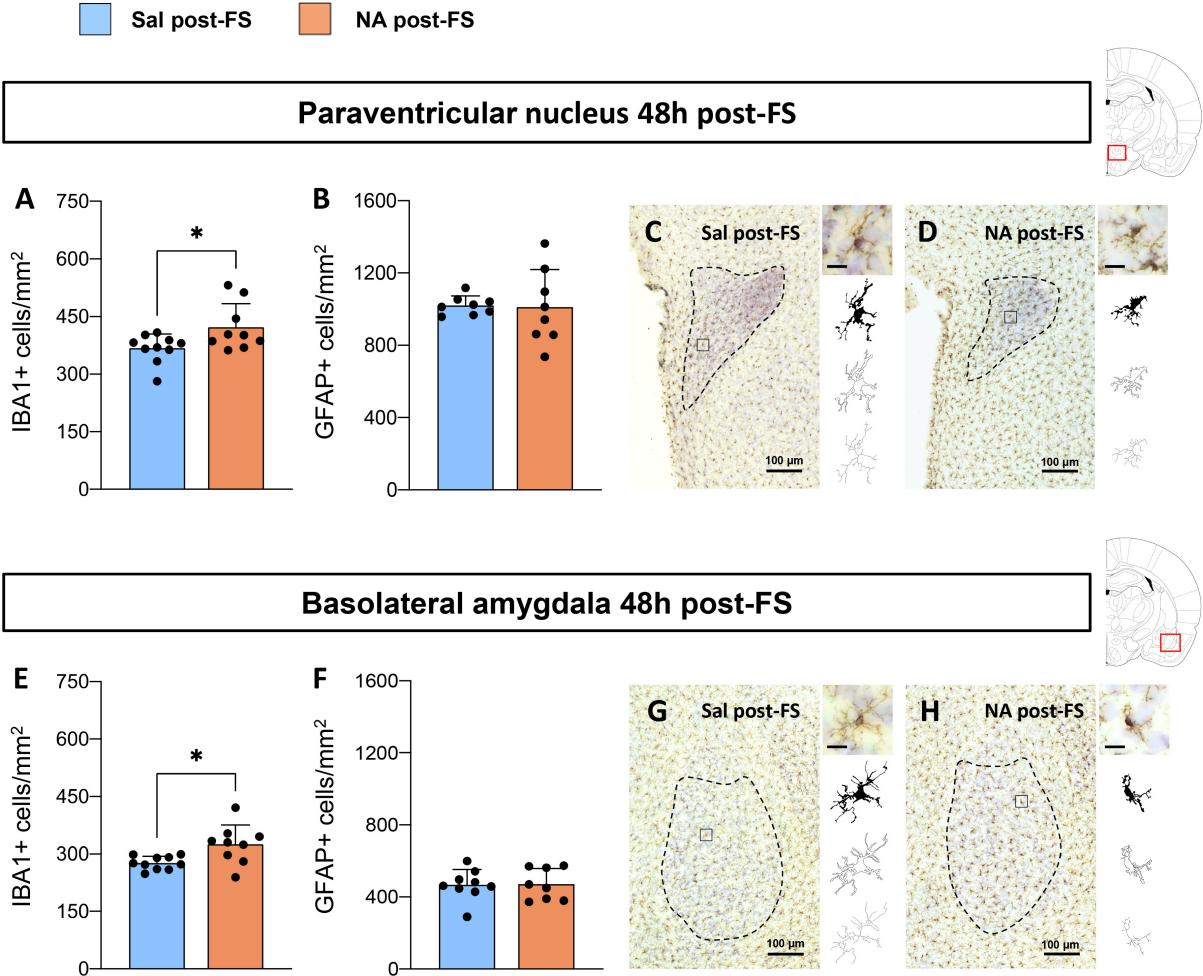
Microglia and astrocytes in the hypothalamic PVN and amygdala after NA-induced neuroinflammation and acute stress. Rats injected with neuraminidase (NA) or saline (Sal) were 3 months later exposed to forced swimming (FS). They were euthanized 48 hours after FS. Coronal brain sections at the level of the paraventricular nucleus (PVN; **A**–**D**) and the basolateral amygdala (BLA; **E**–**H**) were immunostained for IBA1 (microglia; **A**, **E**) and GFAP (astrocytes; **B**, **F**). (**C**, **D**, **G**, **H**) Representative sections of PVN **(C, D)** and BLA **(G, H)** from NA and saline injected and later FS stressed rats, immunostained for IBA1. A representative cell (squared) is shown enlarged on the side along with its shape profiles (filled, outlined, and skeleton shapes) obtained for the subsequent morphological analysis. Scale bars in the enlarged images are 10 μm. The histograms show the mean ± SD and the individual values of n = 7–9 animals per group. *p < 0.05.

**Figure 6:**
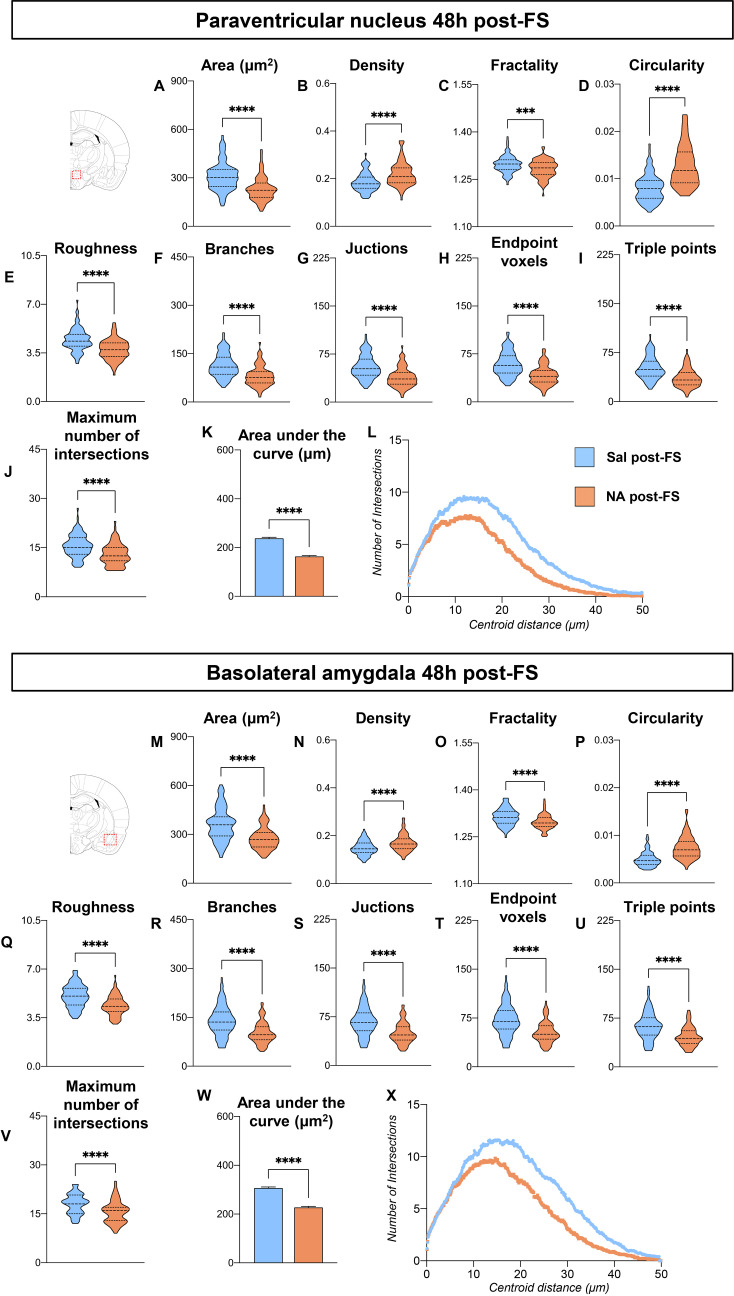
Morphological analysis of microglial cells from rats exposed to NA-induced neuroinflammation and acute stress. Rats injected with neuraminidase (NA) or saline (Sal) were 3 months later exposed to forced swimming (FS). They were euthanized 48 hours after FS. Coronal sections at the level of the paraventricular nucleus (PVN) and the basolateral amygdala (BLA) were immunostained for IBA1 to label microglial cells. Morphological analysis was carried out by three different methods: Fractal analysis **(A–E, M–Q)**, Skeleton analysis **(F–I, R–U)** and Sholl analysis **(J–L, V–X)**. Representative parameters obtained are shown here; additional parameters are available as Supporting information. Data distribution of each parameter is presented as a violin plot, which has been truncated at the maximum and minimum values of each dataset; the dashed line represents the median and the dotted line the quartiles. **(K, W)** The *area under the curve* for the graphs showing *number of intersections* over *distance from the centroid* **(L, X)** was calculated with the results from Sholl analysis. The histograms show the mean ± SD. A total of n = 155–190 cells from PVN and from BLA, sampled from different animals within each experimental group, were analyzed. ***p < 0.001, ****p < 0.0001.

First, the density of resident immune cell populations, microglia and astrocytes, was addressed after acute FS, in the brain areas formerly selected, PVN and basolateral amygdala (BLA). Microglial density (IBA1-positive cells) was increased in both areas 48 hours after FS stress in NA-injected animals compared to saline-injected controls (**Figures 5A, C–E, G, H**; **Supplementary Figure 7**). However, when it comes to astrocytes (GFAP-positive cells), no differences were found between NA and saline groups (**Figures 5B, F**). Therefore, in PVN and BLA, microglia population slightly increased upon acute stress in NA-injected animals compared to saline controls, while astrocytes numbers remained the same. Also, in NA-injected rats microglial cells appeared more intensely stained with IBA1 and less ramified both in PVN (compare **Figures 5C, D**) and BLA (compare **Figures 5G, H**). Morphological differences in microglial cells were further characterized by objective morphological analysis of individual cells (**Figure 6**; **Supplementary Figures 4**, **5**). A total of 28 parameters of the cell morphology were obtained using three different methods: Fractal analysis, Skeleton and Sholl (**Supplementary Figures 4**, **5**); only 10 of them are shown here for the sake of simplicity (**Figure 6**). Because microglial cells differ in their shape amongst different brain regions, PVN and BLA cells were assessed separately.

Microglial cells sampled from NA-injected and FS stressed rats showed reduced area, fractality and roughness, and increased density and circularity (parameters obtained from the fractal analysis), both in PVN (**Figures 6A–E**) and BLA (**Figures 6M–Q**), indicating that cells are smaller, rounder, more compact and less complex. Skeleton analysis, which focuses on complexity of ramifications, revealed reduced number of branches, junctions, endpoint voxels and triple points, denoting less ramified cells both in PVN (**Figures 6F–I**) and BLA (**Figures 6R–U**). Finally, the Sholl method (an alternative way of evaluating cell branches that relies on the intersections of branches with concentric imaginary circles of increasing radius drawn from the cell´s centroid) disclosed graphs of number of intersections vs centroid distance that were skewed down and left in cells sampled from NA × FS rats (**Figures 6L, X**), indicating that ramifications are both less abundant and located closer to the cell´s centroid. The area under the curve obtained from these graphs endorsed a statistically significant difference between NA and saline groups (**Figures 6K, W**), as well as the maximum number of intersections (**Figures 6J, V**), both parameters being reduced in cells of NA injected and FS stressed rats.

In summary, these data indicate that in those rats that experienced NA-induced neuroinflammation, the occurrence of a second neuroinflammatory stimulus, as is the acute stress caused by FS, three months later provokes an exacerbated reaction (evidenced by increased cell density and cell morphology) of microglial cells located in brain structures relevant for the stress response, such as hypothalamic PVN and amygdala.

### 3.4 Complex behavioral profile after acute stress in rats that suffered neuroinflammation three months before

**Figure 7:**
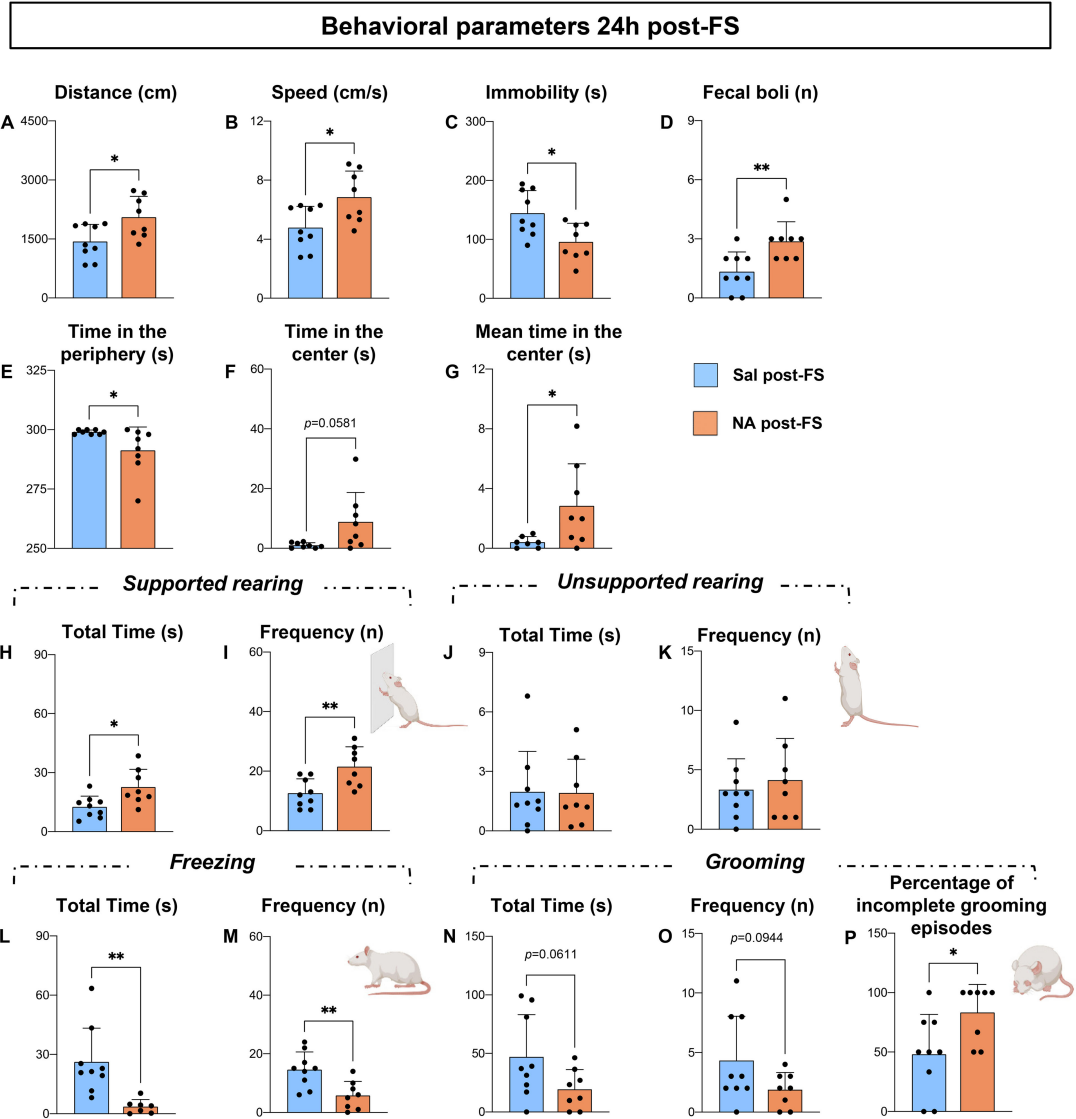
Behavioral evaluation in the open field 24 hours after acute stress. Rats were neuraminidase (NA) or saline (Sal) injected and, 3 months later, exposed to acute stress with forced swimming (FS). Twenty-four hours later the animals performed an open field test for 5 minutes. The parameters measured are related to locomotor behavior **(A–C),** exploration of the arena **(E–G)**, supported rearing **(H, I)**, unsupported rearing **(J, K)**, freezing **(L, M)** and grooming **(N–P)**. **(D)** The number of fecal boli is also presented. Representative parameters are shown here; additional parameters are available as **Supplementary Information**. The histograms show the mean ± SD and the individual values of n = 8–9 animals per group. *p < 0.05, **p < 0.01. *p*-values close to significance (*p* < 0.15) are also shown.

In order to address potential behavioral disturbances in rats exposed to the combined neuroinflammatory and acute stress conditions, we conducted the open field test (OFT). The animals performed the OFT 24 hours after being exposed to the FS stressor (**Figure 7**; **Supplementary Figure 6**). Regarding locomotor activity, the rats ICV injected with NA travelled a longer distance and developed a higher speed, along with reduced immobility, in the open field, compared to those that underwent sham surgery (**Figures 7A–C**), what may suggest hyperactivity. Also, the NA-injected rats produced more fecal boli during the test (**Figure 7D**), suggesting altered stress-reactive or emotional responses within the OFT context. NA-injected rats spent more time in the center of the arena (**Figures 7E–G**); however, center-related measures should not be interpreted as evidence of reduced anxiety given the concomitant hyperlocomotion. Regarding specific behaviors, NA-injected rats showed more supported rearing (**Figures 7H, I**), while similar unsupported rearing (**Figures 7J, K**), than saline injected controls, and less freezing (**Figures 7L, M**), which could be related to the increased locomotor activity. Finally, NA-injected rats displayed a tendency to perform less grooming (**Figures 7N, O**), with significantly increased incomplete grooming episodes (**Figure 7P**), suggesting disruption of normal grooming organization. Therefore, prior neuroinflammation influenced the behavioral response following exposure to an acute stressor. Compared to ICV saline control rats exposed to FS, those rats exposed to the two insults (NA × FS) displayed a complex OFT-derived behavioral profile characterized by hyperactivity and alterations in exploratory, stress-reactive, and risk-assessment-related behaviors.

## 4 Discussion

Neuroinflammatory history determines the response to new inflammatory stimuli. One of the underlying mechanisms relies on microglial cells, which are capable of acquiring immune memory of previous inflammatory events. Thus, microglia acquire a primed phenotype, characterized by exacerbated responses when they are exposed to novel inflammatory stimuli (7, 11, 94). We previously showed that hypothalamic microglia get primed when experimental animals suffer acute severe neuroinflammation, which was provoked by a single ICV injection of the microbial enzyme neuraminidase (NA) (14, 41, 71) Interestingly, this primed state persisted for at least three months (while acute inflammation is largely solved in about two weeks; 88). Thus, exposure of animals to a second stimulus (such as peripheral immune stimulation by administering LPS, a short-term high-fat diet, or a situation of obesity) three months later provoked a heightened activation of hypothalamic microglia and increased neuroinflammation. Moreover, functions depending on hypothalamic regulation, such as body weight and feeding, were altered (14). Therefore, we aimed to investigate further the long-term impact of NA-induced priming on the hypothalamus.

Using a similar two-insults paradigm, here we explored if previous neuroinflammation determines the response to an acute psychological stressor, specifically forced swimming (FS). After six minutes of FS, rats that had experienced severe neuroinflammation three months before showed an increased activation of the HPA axis, evidenced by significantly higher levels of plasma CORT. Such heightened activation concurred with an enhanced and prolonged negative feedback inhibition of the HPA axis (e.g. decreased CRH and AVP gene expression, increased CRH protein accumulation, increased CORT receptor expression in the hypothalamus, and increased endocannabinoids synthesis, measured in periventricular hypothalamus tissue). Moreover, the former NA-induced inflammation also resulted in an enhanced (although modest) inflammatory response after FS, supported by the increased expression of several inflammatory markers (e.g. cytokines, IBA1, inflammasome, and NFκB pathway), some of which were even more evident 48 hours after FS than at a shorter time (12 hours after FS). Such enhanced neuroinflammation was further confirmed by analyzing microglial cells located in PVN and amygdala, which showed a mildly increased cell density along with a cell morphology consistent with a more reactive phenotype. In a previous work, we identified a significant population of IBA1/MHCII−positive cells that were largely confined to the medio-basal hypothalamus (arcuate nucleus and median eminence), suggesting a possible peripheral origin. In fact, they were virtually absent from other hypothalamic regions, including the PVN (14). MHCII, while often upregulated in activated or primed microglia, is not a definitive marker of priming. Given that morphological parameters provide robust quantitative readouts, we therefore focused on microglial morphology to analyze microglial priming. Although IBA1 is not fully microglia-specific, it was selected because it provides robust staining for detailed morphological analysis. Nonetheless, future studies incorporating a broader marker panel, including microglial homeostatic and activation markers such as MHCII, CD68, P2RY12, and TMEM119, will further strengthen phenotypic characterization.

Finally, behavioral alterations in the open field also arose after FS, with an array of symptoms (including hyperactivity, increased time spent in the center, defective grooming, etc.) pointing to a complex behavioral phenotype. The apparent reduction in thigmotaxis observed in NA × FS animals, evidenced by increased time spent in the center of the open field, does not reflect a genuine anxiolytic effect, but rather the emergence of a hyperactive and disinhibited behavioral phenotype. In these animals, heightened locomotor drive combined with impaired risk assessment mechanisms likely override the innate aversion to exposed areas, resulting in prolonged exploration of the center zone. This “false” anxiolytic profile aligns with the concurrent increases in total distance traveled, locomotor speed, supported rearing, and fecal boli production—all of which are indicative of elevated arousal and stress reactivity. Collectively, these findings argue against a true reduction in anxiety and instead point to a dysregulated behavioral state characterized by hyperactivity, heightened stress sensitivity, and diminished environmental filtering.

Overall, these results indicate that the HPA axis is sensitized by a single episode of past neuroinflammation, even when that happened a long time ago (hence, sensitization persisted for at least three months). Enhanced feedback inhibition of the HPA axis along with behavioral alterations after acute stress suggest maladaptation to stress. Priming of microglial cells by the previous NA-provoked neuroinflammation is a potential underlying mechanism for these HPA axis alterations.

A limitation of the present work is the lack of experimental groups free of the acute stressor FS, where one could assess the basal levels of both CORT and inflammation three months after NA or saline injection, without the inflammatory activation triggered by FS itself. For the sake of minimizing the number of animals used, these groups were intentionally omitted, as we considered that the main question of the work (i.e. Are the stress and the inflammatory responses provoked by acute stress conditioned by previous neuroinflammation)? could be addressed by comparing Sal × FS with NA × FS groups.

It has been extensively reported the existence of sex differences in the HPA-axis response to stress as well as in the immune activation triggered by stress (95–98). In general, females are more vulnerable to stress-related psychiatric disorders, while males are more susceptible to the consequences of immune activation early in life (99–101). Furthermore, male and female rodent microglia exhibit differences in their morphology and function, which may explain sex-dependent susceptibility to neurological diseases (95, 102, 103). Mechanisms including differential exposure to hormones along lifespan, a high proportion of immune-related genes located in the X chromosome, differences in epigenetic regulation, or variations intrinsic to microglial cells, may account for the sexual dimorphism described in neuro-immune responses and disease vulnerability (103). Therefore, since this study only used male rats, the results obtained cannot be extrapolated to females, and highlight the need to investigate how past neuroinflammation modulates stress responses specifically in females.

### 4.1 Neuroinflammation provoked by NA induces sensitization of the HPA axis and of the response to acute stress

Different paradigms have been used with the purpose of exploring the interactions between psychological stressors, immune activation and HPA axis dysregulation. Central neuroinflammation may be triggered by acute or chronic psychological stress (16, 76, 104–106), traumatic brain injury (8, 79), cold exposure (107) or microbial infections (74, 108–110), similar to that induced here by the ICV administration of microbial NA (88). Peripheral immune activation, most frequently achieved by the i.p. administration of viral/bacterial mimetic, also impacts on the central nervous system, as it is readily conveyed to the brain by various pathways (circumventricular organs, choroid plexus and vagus nerve; 6, 12, 69, 111, 112). Sensitization of the HPA axis and heightening of the stress response, along with mood disorders, have been reported as a result of inflammation of central or peripheral origin (reviewed in 22, 23, 34, among others, 77, 113, 114) which is consistent with our results. Even so, in some sporadic situations, however, exposure to a first challenge resulted in a blockade or attenuation of the stress response (82, 115). The commonality of all these paradigms lies on an initial stimulus which activates the innate immune response in the brain, with a significant increase of inflammatory mediators, such as cytokines, within the nervous parenchyma. The various outcomes reported in different works may be due to factors such as the nature (immune, psychological, physical, etc.) and intensity of the sensitizing/priming stimulus, the moment of the exposure (e.g. perinatal versus adult exposure), the recurrence of the exposure (chronic versus acute), and the time relapse between exposure to the first sensitizing stimulus and the stressful experience (107, 113, 116–120).

In general, animals exposed to an initial stimulus develop a central inflammatory response that, if intense enough, will drive sensitization/priming. The priming stimulus used here (ICV administration of NA) consists in a central immune challenge that mimics a viral/bacterial brain infection, which recapitulates archetypal stages and features of neuroinflammation along with its subsequent resolution, as animals are allowed to recover for a long period of time (88). Adult central immune activation induced by microbial components is rarely used in paradigms assessing the impact of immune activation on the stress response, thus making this work rather unique. Noteworthy, NA is an enzyme that forms part of some neurotropic microorganisms, such as mumps virus (121) and strains of bacteria producing meningitis (122). Influenza virus also carries NA in its envelope, so NA inhibitors are a widespread flu treatment; while influenza virus mostly targets the respiratory system, some strains are neurotropic, and cases of neurological complications are not unusual (112, 123, 124). Alternatively, peripheral immune activation is widely achieved by i.p. administration of LPS (a bacterial mimetic) or poly(I:C) (a viral mimetic); peripheral inflammation is then readily transmitted to the brain, inducing central inflammation (6). Such immune challenge applied to pregnant dams (maternal immune activation or MIA model) or shortly after birth provokes an immune activation with behavioral consequences in the adult offspring, thus illustrating how acute infections (particularly those occurred in the perinatal period) may imprint altered behavioral responses to challenging situations in the future (125–131). Our results are in line with these observations, as exposure to a second challenge (acute FS) provoked enhanced CORT secretion and behavioral disturbances in rats that had previously experienced neuroinflammation. In neonatal models, sensitization is mostly attributed to inflammatory mediators (mostly cytokines) interfering with neurodevelopment. However, this cannot be argued in our model with adult rats. Microglial priming will be discussed below as a potential mechanism.

A relevant component of HPA axis sensitization is its impact on stereotypic behaviors triggered by acute stress. This was here explored by assessing animals in the open field. Anxiety and depressive-like behaviors are frequently observed after HPA axis sensitization (9, 69, 74, 76, 78, 130, 132–136), although our results do not support such outcomes. While rats that underwent previous neuroinflammation exhibited an array of altered behaviors (more time in the center of the arena, increased supported rearing, less freezing, incomplete grooming, more fecal boli) increased mobility (longer distance traveled and higher speed) was the clearest symptom. NA-treated rats demonstrated increased mobility and exploratory behavior, which may indicate hyperactivity potentially induced by underlying stress dysregulation. Despite showing increased center exploration in the OFT, NA-treated animals exhibited enhanced CORT after FS, suggesting an overreactive physiological response to acute stress. Interestingly, hyperactivity with no anxiety-like behavior in the open field has been described in rats that had been previously (5 weeks earlier) exposed to stress by adolescent social isolation (137), as well as in a mouse model of mucopolysaccharidosis IIIB (138). Increased locomotor activity and rearing behavior after acute stress are recognized as consistent indicators of stress responsivity and CORT secretion (139–141); our results are in line with these reports, although other mood symptoms are difficult to interpret. Interestingly, in a two-stressor paradigm (where escapable or non-escapable tailshock was followed by FS) anxiety symptoms provoked by FS were prevented when the animals had control over the tailshock previously applied (escapable vs non-escapable), thus emphasizing the importance of perceived stress and context in stress outcomes (132).

The pronounced hyperactivity observed in the open field, together with increased exploration of the center area and reduced freezing behavior, is better interpreted as a manifestation of behavioral disinhibition and heightened arousal, rather than a true reduction in anxiety-like behavior. In this context, increased locomotion may represent a dysregulated response to stress (142) potentially driven by neuroinflammation-induced alterations in neural circuits that normally constrain exploratory and defensive behaviors. Despite the increase in central area occupancy, typically associated with reduced anxiety, this behavior likely reflects a disruption in risk assessment mechanisms caused by the hyperactive state, rather than a genuine anxiolytic effect.

Although increased time spent in the center of the open field is traditionally interpreted as indicative of reduced anxiety-like behavior, this parameter must be evaluated cautiously, particularly in the context of altered locomotor activity. In our study, rats previously subjected to neuroinflammation (NA × FS group) displayed a clear hyperactive phenotype, characterized by significantly increased total distance traveled, higher speed, and reduced immobility. These changes are consistent with a state of generalized behavioral overactivation, which can interfere with innate avoidance responses such as thigmotaxis (143). Under normal conditions, rodents avoid the exposed center of the arena due to its anxiogenic properties; however, in hyperactive animals, the drive to explore may dominate over risk assessment mechanisms, leading to increased entries and prolonged occupancy in the center zone. This disinhibition does not necessarily reflect reduced anxiety, but rather a disruption in the integration of environmental salience and behavioral control.

Thus, the increased center time should be interpreted as a secondary consequence of hyperlocomotion and impaired inhibitory control, rather than a true anxiolytic effect. This interpretation is supported by the broader ethological profile observed in the OFT, including reduced freezing, increased supported rearing, altered grooming structure, and increased fecal boli, which together point to changes in exploratory activity, stress reactivity, risk assessment, and behavioral organization. Importantly, these variables should be considered together with locomotor parameters, rather than interpreted in isolation, since the combination of increased locomotion, reduced immobility/freezing, and altered ethological responses is consistent with a phenotype of behavioral overactivation and disinhibition. In this framework, the OFT provides an integrated behavioral readout consistent with an altered response to acute stress after prior neuroinflammatory challenge. Future studies including additional behavioral paradigms, preferably locomotion-independent or less locomotion-confounded, could further delineate specific anxiety-like, fear-related, or stress-coping components of this phenotype.

The apparent dissociation between elevated CORT levels and behaviors typically interpreted as indicative of reduced anxiety in NA-treated animals suggests that prior neuroinflammation may sensitize the HPA axis, leading to maladaptive stress reactivity. In this scenario, hyperactivity may emerge as a consequence of impaired feedback control within the HPA axis, which hinders the ability to generate adaptive behavioral responses to stress. Indeed, the hyperactivity and increased arousal seen in NA-treated rats resemble some features of PTSD, where disinhibition and heightened responsivity to environmental cues are commonly reported (144, 145). Interestingly, PTSD patients also exhibit enhanced HPA axis negative feedback (51), a pattern similarly observed in our NA-treated animals. While this phenotypic parallel is compelling, additional behavioral paradigms—particularly those designed to assess fear learning, avoidance behavior, and stress habituation—will be essential to determine the extent to which this model recapitulates key aspects of PTSD.

These findings highlight the complex interplay between neuroinflammation, hyperactivity, and stress responses, emphasizing the need for further research to understand the underlying neurobiological mechanisms and the implications for stress-related hyperactivity disorders. In this sense, in an interesting previous work the blockade of CORT synthesis before foot shock resulted in a blunted CORT increase, but had no impact on the increased grooming or rearing behaviors. Also, photo-stimulation and/or photo-inhibition of PVN CRH neurons led to conclude that CRH neurons orchestrate a complex repertoire of behaviors triggered after acute stress (e.g grooming, rearing and walking), some of which are CORT independent (140).

### 4.2 Microglia and cytokines as drivers of HPA axis sensitization

As mentioned before, a variety of challenges are able to induce neuroinflammation, increasing the production of cytokines and other inflammatory mediators within the brain. Among them, cytokines like IL6, IL1β and TNFα, which are mostly secreted by activated microglia, have been proposed to play a role in sensitization of the HPA axis. In maternal immune activation models, cytokines have been implicated in altered neurodevelopment, with subsequent consequences in stress response, behavior and cognition (126, 127, 146). In adult rats, a single injection of IL1 was able to induce long lasting changes in the HPA axis and its sensitization to stress (147, 148). Other authors reported similar outcomes when using TNFα (149). The ICV injection of NA provokes an acute increase in the expression of IL6, IL1β and TNFα in the hypothalamus (150, 151), so these cytokines could also mediate HPA sensitization in this model. In the nervous parenchyma activated microglia are not only the main source of cytokines, but also the core of immune priming within the brain. Because glucocorticoids levels are also acutely increased by the majority of stressors, they have been as well proposed as mediators of microglial priming (102).

Here, priming of microglial cells in PVN and amygdala was evidenced by amplified morphological changes after exposure of rats to acute FS. The role of primed microglia on HPA axis sensitization is highlighted by experiments where, by transient pharmacological inhibition of CSF1R receptor, the population of microglia was depleted and later replaced. This strategy reverted microglial alterations and behavioral dysfunctions that had been previously induced by maternal immune activation (129) or by chronic stress in a repeated social defeat model (78). Moreover, the depressive-like behavior induced by chronic unpredictable stress in rodents could be prevented by the previous blockade of microglial activation with minocycline (57). Despite evidence suggesting the relevance of primed microglia in the exacerbated responses observed in NA-treated rats exposed to stress, the lack of specific microglia depletion/repopulation experiment limits the present study’s ability to definitively establish a causal link. Thus, our findings should be interpreted as supporting a strong association between microglial priming and these outcomes. Future experiments should address this critical issue.

Although immune and stress responses are frequently simultaneous and somehow intertwined, some evidence supports different mechanisms for microglial priming and for CORT response sensitization. Thus, central IL1β was sufficient to sensitize both the cytokine (IL1β) response and the CORT response to a subsequent immune stimulus. Nonetheless, administration of an IL1 receptor antagonist was able to block sensitization of the cytokine (IL1β) response, but did not affect CORT response sensitization (152). Furthermore, in an elegant experimental design where sensitization was induced by repeated social defeat in mice, Weber et al. showed that microglial elimination/repopulation did not affect the anxious response to acute stress, indicating that the behavioral component of the stress response was independent of microglial priming. On the contrary, microglial reactivity to a peripheral immune challenge (LPS) was reverted with the same strategy, suggesting different underlying mechanisms for behavioral and immune sensitization. These authors inferred that other components apart from microglia are involved in chronic stress sensitization, and provided evidence that neurons and splenic monocytes (which are recruited to the brain upon acute stress) may also retain memory of past inflammatory events and therefore determine the response to acute stress (78). The difficulties encountered to discriminate the effects mediated by glucocorticoids from those dependent on cytokines might be due to the fact that both the stress and the inflammatory responses seem to trigger a common stereotypic innate cellular defense response (153).

### 4.3 Microglia and cytokines as mediators in HPA axis hyperactivation

In order to assess the degree of inflammation triggered after acute FS, we explored the expression levels of several inflammatory markers in periventricular hypothalamus (where PVN is located) and amygdala; the ratio of phosphorylated to non-phosphorylated NF-κB (indicative of activation of the pathway) was also quantified in hypothalamus. Although various of these markers were enhanced in NA-treated rats, in some cases depending on the brain region (hypothalamus or amygdala) or post-stress time (12 or 48 hours), the differences between NA- and saline-injected rats were not as generalized (among all markers used) or evident as would be expected. It needs to be considered, though, that saline-injected rats were also exposed to FS, and therefore inflammation in this control group is expected to be above basal levels as well. Some interesting observations are: 1) enhanced expression of relevant markers such as IBA1 and inflammasome NLRP3 was only observed 48 hours after acute stress, in both hypothalamus or amygdala; 2) cytokines expression (TNFα, IL1β and IL6) was not noticeably enhanced in NA-treated rats, with the exception of IL1β in hypothalamus and only at 12 hours post-FS; 3) moreover, in NA-group, IL6 expression not only did not increase, but was even downregulated in hypothalamus 48 hours after FS. When evaluating these results, it is necessary to consider that in the hypothalamus/amygdala of these animals might be concurring local pro-inflammatory mediators, induced by the acute FS stress, along with the anti-inflammatory effects of potentially elevated plasma CORT (although we cannot confirm such elevation, as previously stated), thus depicting a complex scenario difficult to interpret (154). Overall, our results seem to indicate that, although FS induces inflammatory activation, the anti-inflammatory effects of CORT cannot completely override the strong inflammatory reaction triggered by the acute stressor, especially when such reaction is amplified by prior NA-induced inflammation. Thus, the immunosuppressive properties of glucocorticoids could explain the downregulation of IL6 in NA-treated rats (48 hours post-FS). However, this argument remains a hypothesis, as our data only demonstrate elevated CORT levels immediately after FS (10–30 min), but not at later times.

It has been consistently described that immune activation widely overlaps with the activation of the stress response. The same occurred in our experimental paradigm, where the exposure of rats to acute FS stress elicited both CORT secretion (within minutes post-FS) and cytokine expression. Moreover, in those rats that underwent NA-induced inflammation in the past, CORT plasma levels and inflammatory markers were both heightened, as well as the reactive morphology of microglial cells, compared to saline-treated rats. Therefore, we may speculate that, upon acute FS stress, pro-inflammatory cytokines released by primed hypothalamic microglia (particularly IL1β, as it was the only cytokine which expression was clearly enhanced) could facilitate CRH/AVP secretion, leading to increased plasma CORT. In fact, the ability of IL1β and other cytokines (mainly TNFα and IL6) to directly stimulate CRH neurons in PVN resulting in a burst of plasma CORT has been previously established (44, 49, 65, 66, 155). On the other hand, CORT itself can limit microglial activation, what could explain the moderate (instead of substantial) increase in the inflammatory markers observed in NA-treated rats (where CORT levels immediately after FS were strikingly enhanced) as well as the suppression of IL6 expression (42, 154). Remarkably, noradrenalin binding to β-adrenergic receptors on microglial cells has been demonstrated to induce IL1β secretion by these cells, thus unveiling a mechanism linking stress related neurohormones with microglial activation (104, 111, 136, 156, 157).

In addition to cytokines, microglial cells could yet modulate CRH neurons activity during acute stress by other mechanisms. Microglia sense, respond to, and regulate neuronal activity through various signaling pathways, such as ATP/ADP binding to P2Y12 receptors or noradrenalin binding to β-adrenergic receptors on microglial cells. Then, microglial processes can engage with neuronal axons and dendrites, thus modulating synaptic transmission, neuronal firing and overall activity of neuronal circuits (21). Moreover, it has been recently described that microglial cells can regulate the activity and the survival of neurons through novel specialized purinergic junctions stablished between microglia and neuronal soma (158).

### 4.4 Sensitization of the HPA axis in adult rats persists over time

Another aspect that influences the outcome in two-hit paradigms is the time interval between the two insults. In the present work animals were allowed to recover from NA-induced neuroinflammation for a long period of time before being exposed to the acute FS stressor. Thus, at the moment of applying FS the inflammatory status in the brain was (or was very close to) basal. In fact, three months after NA injection microglial morphology in hypothalamus and amygdala, as well as most markers of inflammation, are similar to those observed in sham injected controls (**Supplementary Figure 3**; 14, 75). Therefore, the inflammatory activation observed here after FS was provoked by the stressor itself, without confounding effects of the previous NA-induced inflammation, which could act, however, as a FS-response modifier. Thus, this design allows to evaluate how the consequences (in terms of inflammatory activation, HPA axis activation, and behavioral response) of a sole episode of acute psychological stress can be modified by a single severe neuroinflammatory event occurred in the distant past (here, three months earlier). Although in most studies carried out with adult animals the two insults were applied closer in time, medium-term sensitization/priming of the acute stress response has been previously reported, with intervals between stimuli of 3 to 4 weeks (78, 148, 149). However, scarce reports explore microglial priming lingering beyond this time frame; among them, some studies have shown that sensitization may fade away with time (147) while one particular work describes microglial priming induced by peripheral immune stimulation lasting for at least six months (13). Importantly, persistent microglial priming could explain mood disorders arising several months after mild TBI in patients that were reported asymptomatic two weeks after injury (133).

Long-term sensitization has been particularly explored during development, where sensitization was induced either by maternal immune activation or challenging offspring at the first days of life. Immune activation using bacterial or viral mimetics during this vulnerable neurodevelopmental period programs both the immune and the neuroendocrine systems throughout life (116, 159). Adult offspring are prone to anxiety and depressive like behaviors (130, 131, 135) or may present memory deficits (68, 125), which become more evident upon exposure to acute stress or infection (68, 119, 125, 160, 161). Microglial priming, which persist through adulthood, has been shown to underlie these behavioral symptoms (68, 70, 129, 135). According to our results and those of others, persistent microglial priming also occurs when adult animals are exposed to a centrally (14, 71) or peripherally administered (13) immune challenge, indicating that it is not restricted to neurodevelopmental stages. Although the precise mechanism is not yet clear, it has been suggested that previous immune activation decreases basal glucocorticoid negative feedback inhibition of the HPA axis, thus increasing HPA responsiveness upon acute stress exposure (119). Moreover, persistent epigenetic modifications induced by inflammatory activation of microglial cells have been proposed to underlie immune memory; the slow turnover of these cells may contribute to the persistence of epigenetic modifications, and therefore priming, over time (12, 13).

### 4.5 HPA axis sensitization is associated with enhanced negative feedback after acute stress

The CORT response after acute stress is rapidly counteracted by various negative feedback mechanisms, mostly mediated by CORT, that efficiently operate for plasma CORT levels to return to baseline. Wistar Kyoto rats, which seem to be particularly sensitive to acute stress, showed a robust CORT response after 15 min FS, with CORT plasma levels returning to basal levels about 135 min after FS (162). Basal levels are usually recovered faster, and it depends on factors such as the type and the duration of the acute stressor, animal strain and sex. Adult Wistar rats (the strain used here) previously sensitized by LPS injection showed increased CORT response after 30 min restraint stress, with a return to baseline 90 min post-stress (160). Restoration of baseline CORT levels indicates the efficiency of the feedback regulation of the HPA axis. However, as is our case, monitoring the progression of CORT levels at different times post-stress is not always feasible. Here we measured an initial burst in CORT plasma immediately after FS (10–30 min), and the next time point monitored was 12 hours post-stress, moment when NA treated rats showed CORT plasma levels lower than normal (27.5 ± 19.7 ng/ml). This observation could suggest a heightened negative feedback regulation (leading to diminished CORT levels hours after FS) of the HPA axis in NA treated rats due to the previous increased burst of CORT elicited by acute stress.

In line with this, evidence of enhanced feedback inhibition (namely decreased mRNA levels of CRH and AVP, increased CRH protein, and increased endocannabinoids enzymes in hypothalamus) in NA treated rats was observed 12 hours after FS and, remarkably, also 48 hours after FS. However, because data on CORT levels 48 hours after FS are unavailable, no definitive conclusion can be drawn regarding the HPA axis activity at this time point.

Local synthesis of endocannabinoids in the PVN provides a fast (within 10–15 min after stimulus) non-genomic feedback mechanism, independent of classical CORT nuclear receptors (GR/NR3C1 and mineralocorticoid receptor, MR/NR3C2) (92, 163, 164). We observed increased expression of endocannabinoid synthesis enzymes in NA injected rats even 12 hours after FS, compared to non-primed saline controls suggesting the persistence of molecular changes related to this fast feedback mechanism hours after the acute stress. It is worth remembering that plasma CORT levels 12 hours post-FS were notably reduced in NA-treated rats.

Besides, the expression of the GR/NR3C1 receptor in the hypothalamus of NA-treated rats was increased 48 hours post-stress, compared to saline × FS stressed rats. GR and MR receptors are essential to maintaining the sensitivity of the HPA axis, as well as of other brain regions such as the hippocampus or the amygdala, to glucocorticoids. Targeted deletion of GR in the PVN resulted in hyperactivation of the axis (165). Decreased negative feedback sensitivity to CORT and reduction of GR expression, along with increased HPA responsiveness to stress, were observed in adult rats exposed to neonatal immune activation (119). Also, the increased endocrine response to acute stress observed in the Wistar–Kyoto rat was attributed to a reduced sensitivity of GR that provide negative feedback to HPA axis (162). Thus, reduced expression and/or sensitivity of GR receptors in the HPA axis has been associated to reduced feedback inhibition by CORT and dysregulation of the axis. Conversely, our results show increased mRNA expression of GR in the hypothalamus of NA treated rats 48 hours after acute stress, what is coherent with other evidence (e.g. reduced CRH and AVP mRNA levels, accumulation of CRH protein) of enhanced negative feedback.

But factors other than GR expression determine axis sensitivity. Recently it has been unveiled that the co-chaperone FKBP5 is a master regulator of the HPA axis sensitivity to CORT, and polymorphisms in its gene has been associated to psychiatric disorders in humans (166, 167). FKBP5 interaction with GR limits ligand binding and translocation to the nucleus, thus acting as a negative regulator of GR receptors (168). FKBP5 deficiency in CRH neurons of the PVN results in blunted HPA axis response to stress due to increased GR sensitivity (60). A similar outcome was observed when FKBP5 knock down was specifically targeted to POMC-expressing corticotrope cells in the pituitary gland, which plays a key role in GR-mediated negative feedback regulation of the axis (169). Moreover, FKBP5 expression is under regulation of the other glucocorticoid receptor, the MR receptor, leading to modifications of GR sensitivity to glucocorticoids during acute stress (59). To explore if FKBP5 was playing a role in the enhanced negative feedback we did observe in NA treated rats, we measured its mRNA expression after acute stress in our two-hit model; however, we did not find any difference compared to saline injected controls. Therefore, our results suggest that the enhanced negative feedback in NA treated rats depends, at least partially, on increased GR expression and endocannabinoids.

Overall, these results suggest that, after exposure to FS, the initial enhanced activation of the HPA axis in NA treated rats is followed by a sustained feedback inhibition of the axis. A more comprehensive assessment of axis activity, including plasma levels of CORT at later times, would be required to reach more conclusive results.

## 5 Conclusions

This work shows that a single event of acute neuroinflammation can induce changes in the hypothalamus that endure in time and, more importantly, determine the activation of the HPA axis to future acute stressors, as well as the behavioral response deployed. Moreover, evidence shows that priming of hypothalamic (and amygdala) microglial cells could be, at least partially, responsible for HPA axis response sensitization. Further work should explore the specific nature of those long-lasting alterations (microglia depletion strategies should help to confirm the pivotal role of primed microglia on HPA axis sensitization), aside from the potential impact of NA-induced neuroinflammation on other brain structures. Besides, parallel studies carried out with female rats are needed, given the sex differences described in stress responses and HPA-axis function.

In situations of brain infections or damage, the hypothalamus is an understudied region. However, it seems to be particularly vulnerable to brain insults (81). Given the utmost importance of the hypothalamus in regulating basic autonomic functions as well as behavior, our results highlight the importance of long-term monitoring of neuroendocrine systems and mental health in individuals that had suffered severe neuroinflammation of various origins (77, 113, 133, 170–173). Interestingly, strategies aimed at reprogramming microglial priming in laboratory animals have proven effective in reverting behavioral symptoms (9, 78, 106, 129, 130).

## Data Availability

The raw data supporting the conclusions of this article will be made available by the authors, without undue reservation.
