# Supplementary material for: Persistent priming of hypothalamic microglia is associated with sensitization of the hypothalamic-pituitary-adrenal axis to acute stress, hyperactivity and behavioral response disruption in male rats
Source: Front Immunol. 2026 Jun 30;17:1828445. doi: 10.3389/fimmu.2026.1828445 (PMC13364640; doi:10.3389/fimmu.2026.1828445)
Supplement: Supplementary file 1 [file Presentation1.pdf]

Supplementary Methods

2.3 Intracerebroventricular (ICV) injection

Rats received an ICV injection of neuraminidase (NA) to induce acute neuroinflammation, as previously described {Granados-Duran, 2015 #930}. They were anesthetized with a solution of ketamine/xylazine (80 and 12 mg/kg respectively; Sigma Aldrich) and placed in a stereotaxic frame. The skin covering the skull was shaved and cut along the sagittal midline with a scalpel to expose the skull. After locating the Bregma, a drill was employed to make an opening in the skull at coordinates corresponding to the right lateral ventricle: 0.5 mm posterior and 1.4 mm lateral from Bregma. A single dose of NA from *Clostridium perfringens* (Sigma-Aldrich, N3001) dissolved in sterile 0.9% saline solution (500 mU in 20 μL) was administered at a depth of 3.5 mm beneath the dura mater. The infusion was performed using a pump at a rate of 2 μL/minute over a 10-minute period. Control animals received an equivalent volume of sterile saline solution. Once finished the infusion the skin was sutured with surgical staples and cleaned with povidone. Animals were monitored during recovery from anesthesia for signs of pain or distress. Subsequently, they were returned to their original housing conditions.

2.6 Sacrifice and tissue sampling

Rats were deeply anesthetized and blood samples were collected from the heart using a syringe. Then, rats were transcardially perfused with cold saline containing 10 IU/mL of heparin to clear the blood from tissues. Afterwards, the brain was removed and divided along the midline. The right hemisphere, designated for histological studies, was immersed in 4% paraformaldehyde at 4 °C overnight, and then transferred to phosphate-buffered saline (PBS) containing 0.05% sodium azide for storage at 4 °C. The left hemisphere, intended for either RNA or protein extraction, was snap-frozen in dry ice and stored at −80 °C.

The brain regions of interest for RNA and protein extraction were microdissected as follows: the half brains were retrieved from the freezer and placed in dry ice. A rat brain matrix (RWD, 800-00147-00) and high-profile stainless-steel microtome blades were also dry ice cold. Each brain was placed in the matrix and allowed to warm only until soft enough for the blade to cut across. Coronal 2 mm thick pieces were obtained and kept frozen. Those slices of tissue containing the regions of interest (periventricular hypothalamus and amygdala) were retrieved from the matrix and further dissected using a 1 mm tissue biopsy punch, maintaining the tissue frozen during all the procedure. The microdissected samples obtained were 1) placed in RNAse-free tubes and processed for RNA isolation, or 2) placed in low protein binding tubes and processed for protein extraction. The dissected samples were stored at -80 °C until further processing.

2.7 Immunohistochemistry

The brains destined to histological studies were sectioned using a vibratome (Leica VT1000S) to obtain 40-μm coronal sections, which were organized into serial collections and stored in PBS. Sections containing the basolateral amygdala (BLA) and the paraventricular nucleus (PVN) of the hypothalamus were selected for immunohistochemistry (between − 1.5 mm and − 2.0 mm from Bregma approximately).

The following primary antibodies were used for immunostainings: anti-ionized calcium-binding adapter molecule 1 (IBA1), to stain microglia, anti-glial fibrillary acidic protein (GFAP), a marker of astrocytes, and anti-corticotropin releasing hormone (CRH), a PVN neuropeptide.

For IBA1 and GFAP immunostaining free floating sections were washed with PBS and incubated in PBT solution (0.3% bovine serum albumin and 0.3% Triton X-100 in PBS pH 7.3) to block the non-specific binding sites. Primary antibodies (rabbit polyclonal anti-IBA1 1:500, Wako 019-19741; rabbit polyclonal anti-GFAP 1:10000, Dako G9269) were incubated at 4 °C overnight. After PBS washes, sections were incubated with a secondary biotinylated antibody (goat anti-rabbit 1:1000, Pierce) during 2 hours. ExtrAvidin®-Peroxidase (1:1000, Sigma-Aldrich E2886) was used to detect the secondary biotinylated antibodies (1-hour incubation). Peroxidase was visualized incubating the sections during 10 minutes with 0.05% diaminobenzidine (DAB) and 0.03% hydrogen peroxide in PBS. After washes with PBS, the sections were mounted onto gelatin-coated slides, air dried, and counterstained with 0.1% toluidine blue. Then they were dehydrated, cleared in xylene, and coverslipped with Eukitt mounting medium.

For CRH and IBA1 double fluorescent immunostaining, the primary antibodies used were rabbit polyclonal anti-CRH (1:5000, BMA Biomedicals T-4037) and goat polyclonal anti-IBA1 (1:700, Abcam ab5076). As secondary antibodies, donkey anti-rabbit Alexa 488 and donkey anti-goat Alexa 568 (1:1000, Thermo-Fisher A21206 and A11057, respectively) were employed. A 20-minutes wash with PBS containing DAPI (0.2 µg/ml, Sigma-Aldrich D9542) was employed to stain cellular nuclei. The sections were washed with PBS, mounted onto gelatin-coated slides and coverslipped using the anti-fading agent Mowiol 4-88 (Calbiochem/EMD Chemicals).

As a negative control for immunohistochemistry, primary antibodies were omitted.

2.15 Western blot analysis

Using 4-12% polyacrylamide gradient gels Criterion™ XT Bis-Tris Protein Gel (Bio-Rad Laboratories, 3450124), 30 µl (≈ 50 µg) of each protein extract were loaded and separated. After electrophoresis, gels were transferred to nitrocellulose membranes (Bio-Rad Laboratories, 1620115) and stained with Ponceau red in order to confirm equivalent protein loading across the lanes. The membranes were saturated with TBS-T (50 mM Tris-HCl pH 7.6, 200 mM NaCl, and 0.1% Tween 20) containing 2% albumin fraction V from bovine serum (Roche, 9048-46-8) during 1 hour at room temperature. The following primary antibodies were incubated overnight at 4 °C: rabbit polyclonal anti-NAPE-PLD (1:1000, Abcam 95397); rabbit polyclonal anti-DAGLα (1:100, Biorbyt 156533); rabbit polyclonal anti-DAGLβ (1:100, Biorbyt 182976); rabbit polyclonal anti-NF-κB (1:500, Cell Signaling 8242S); rabbit polyclonal anti-NF-κB (Ser536) phosphorylated (1:1000, Cell Signaling 3033S); rabbit polyclonal anti-CRH (1:1000, BMA Biomedicals T-4037). Primary antibodies were incubated with the membranes. As a reference protein, mouse polyclonal anti-γ-adaptin 1:2000 (BD Biosciences 610385) was used. After several washes with TBS-T and 1% Tween-20, the membranes were incubated for 1 hour at room temperature with HRP-conjugated anti-rabbit or anti-mouse IgG secondary antibodies (Promega, Madison, WI, USA), diluted 1:10,000. Following washes in TBS-T, the membranes were incubated during 1 minute with the Western Blotting Luminol Reagent kit (Santa Cruz Biotechnology, CA, USA). Protein bands were detected by chemiluminescence using a ChemiDoc™ MP Imaging System (Bio-Rad, Barcelona, Spain) and quantified by densitometric analysis with the free software FIJI. The results were expressed as the ratio of target protein band to γ-adaptin band or, in the case of NF-κB, the ratio of phosphorylated NF-κB to total NF-κB.
