# Supplementary material for: Persistent priming of hypothalamic microglia is associated with sensitization of the hypothalamic-pituitary-adrenal axis to acute stress, hyperactivity and behavioral response disruption in male rats
Source: Front Immunol. 2026 Jun 30;17:1828445. doi: 10.3389/fimmu.2026.1828445 (PMC13364640; doi:10.3389/fimmu.2026.1828445)
Supplement: Supplementary file 2 [file Table1.pdf]

Table S1. Sequence of the primers used for qPCR

Gene	*Forward (5´>3´)*	*Reverse (5´>3´)*	*Size*	*Accession #*
**CRH**	ATTGCCCACGCTTAGTTTCT	GGGTGACTTCCATCTGCTTT	108	NM_031019.2
**AVP**	AACACTACGCTCTCTGCTTGC	CTCTTGGGCAGTTCTGGAAG	76	NM_016992.2
**OXT**	CTTGGCCTACTGGCTCTGAC	GTCCGCAGGGAAGACACTT	106	M25649.1
**NR3C1**	ATGTTAGGTGGGCGTCAAGT	AGCAGGGTCATTTGGTCATC	95	M14053.1
**NR3C2**	GGAAACCAAAGGCTACCACA	GCAGGTCCAAGTGAAGAACG	99	M36074.1
**FKBP5**	TACCTCCTTGTCAGCGTGTG	ACCTCCCAGAGTTTGCCTTT	120	NM_001012174.2
**IL1β**	GCCAACAAGTGGTATTCTCC	CCGTCTTTCATCACACAGGA	118	NM_031512.2
**TNFα**	CAAGGAGGAGAAGTTCCCA	TTGGTGGTTTGCTACGACG	124	NM_012675.3
**IL6**	AGTTGCCTTCTTGGGACTGA	ACTGGTCTGTTGTGGGTGGT	102	M26744.1
**IBA1**	GGATCAACAAGCACTTCCTC	CTCCAGCATTCGCTTCAA	149	NM_017166.3
**NLRP3**	GGGACTCAAGCTCCTCTGTG	GGCTCTGGTTATGGGTCAGA	131	NM_001191642.1
**MHC II**	GCTGGTCTGTTTCATCAGCA	GCGTCACAGTGCAGGAGTAA	185	NM_198741.2
**TLR4**	GCTGCCAACATCATCCAG	CTCAGCAAGGACTTCTCCAC	185	NM_019178.1
**GAPDH**	TGGAGTCTACTGGCGTCTTC	CTAAGCAGTTGGTGGTGCAG	186	NM_017008.4
