# Supplementary material for: Persistent priming of hypothalamic microglia is associated with sensitization of the hypothalamic-pituitary-adrenal axis to acute stress, hyperactivity and behavioral response disruption in male rats
Source: Front Immunol. 2026 Jun 30;17:1828445. doi: 10.3389/fimmu.2026.1828445 (PMC13364640; doi:10.3389/fimmu.2026.1828445)
Supplement: Supplementary file 3 [file Image1.pdf]

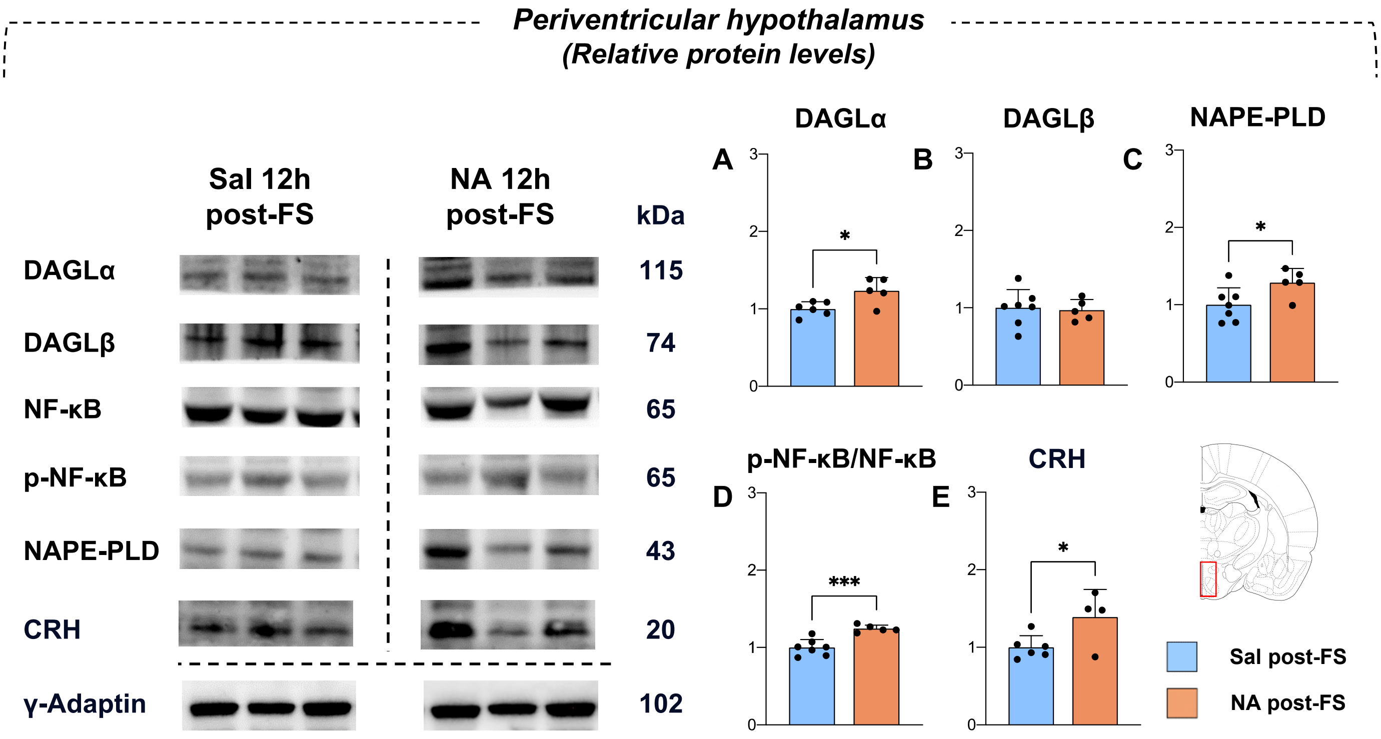

Figure S1. Western blot analysis of hypothalamic protein levels 12 hours after acute stress for endocannabinoid system enzymes, neuroinflammatory proteins and CRH. Rats were injected with neuraminidase (NA) or saline (Sal) and 3 months later exposed to forced swimming (FS). They were euthanized 12 hours after FS for tissue sampling (periventricular hypothalamus). Protein expression was measured by western blot and expressed relative to the level of γ-adaptin. (**A**, **B**, **C**) Key enzymes from the endocannabinoid system (DAGLα, DAGLβ and NAPE-PLD). (**D**) Ratio between phosphorylated (p-) and non-phosphorylated NF-κB protein. (**E**) Corticotropin releasing hormone (CRH) neuropeptide. Histograms show the mean ± SD and the individual values of n = 4-7 animals. *$p < 0.05$, ***$p < 0.001$.
