# Supplementary figures and images for: Persistent priming of hypothalamic microglia is associated with sensitization of the hypothalamic-pituitary-adrenal axis to acute stress, hyperactivity and behavioral response disruption in male rats
Source: Front Immunol. 2026 Jun 30;17:1828445. doi: 10.3389/fimmu.2026.1828445 (PMC13364640; doi:10.3389/fimmu.2026.1828445)

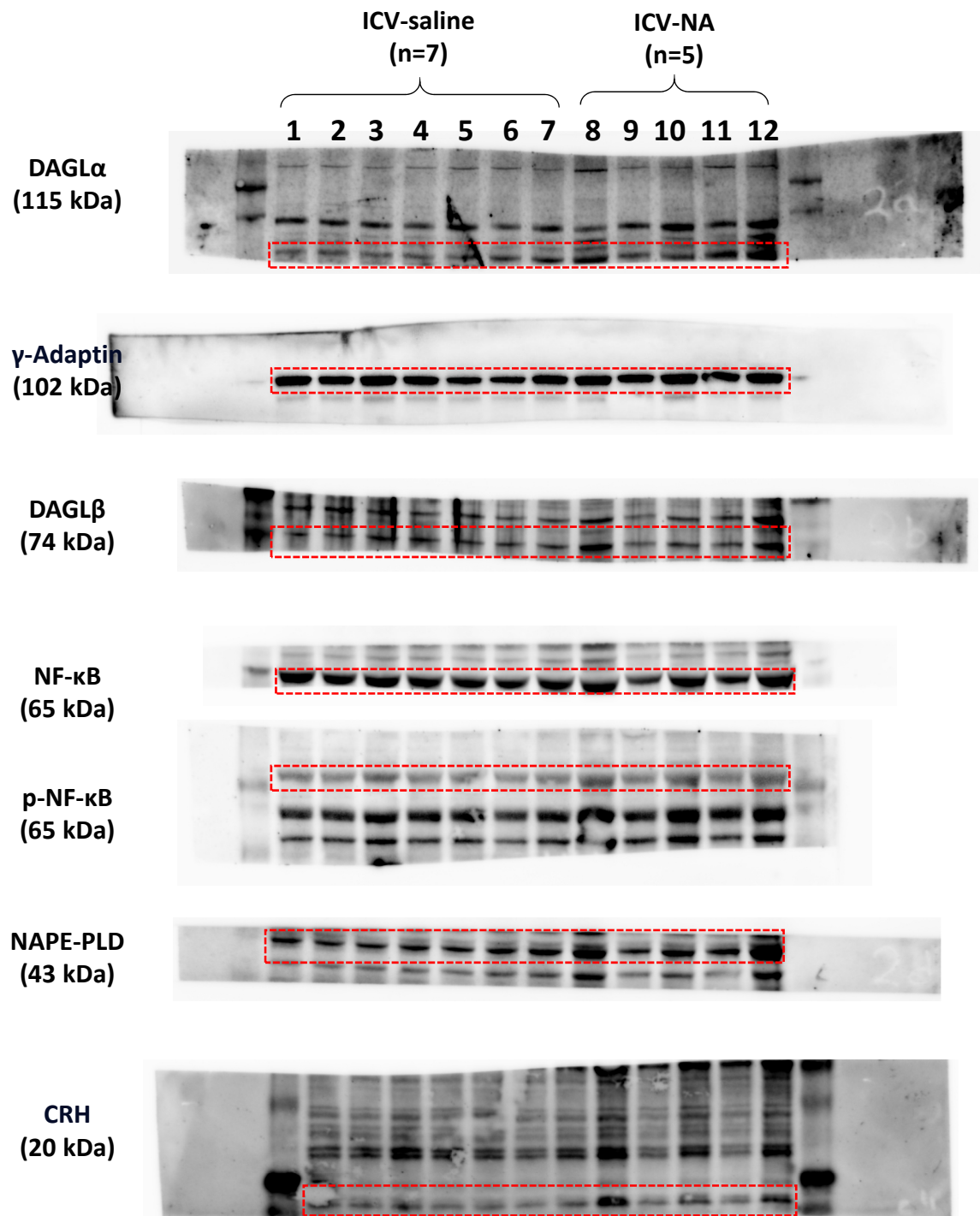

Figure S2. Western blot images of chemiluminescent reaction exposures

Supplement: Supplementary file 4 [file Image2.pdf]
