# Supplementary material for: Persistent priming of hypothalamic microglia is associated with sensitization of the hypothalamic-pituitary-adrenal axis to acute stress, hyperactivity and behavioral response disruption in male rats
Source: Front Immunol. 2026 Jun 30;17:1828445. doi: 10.3389/fimmu.2026.1828445 (PMC13364640; doi:10.3389/fimmu.2026.1828445)
Supplement: Supplementary file 5 [file Image3.pdf]

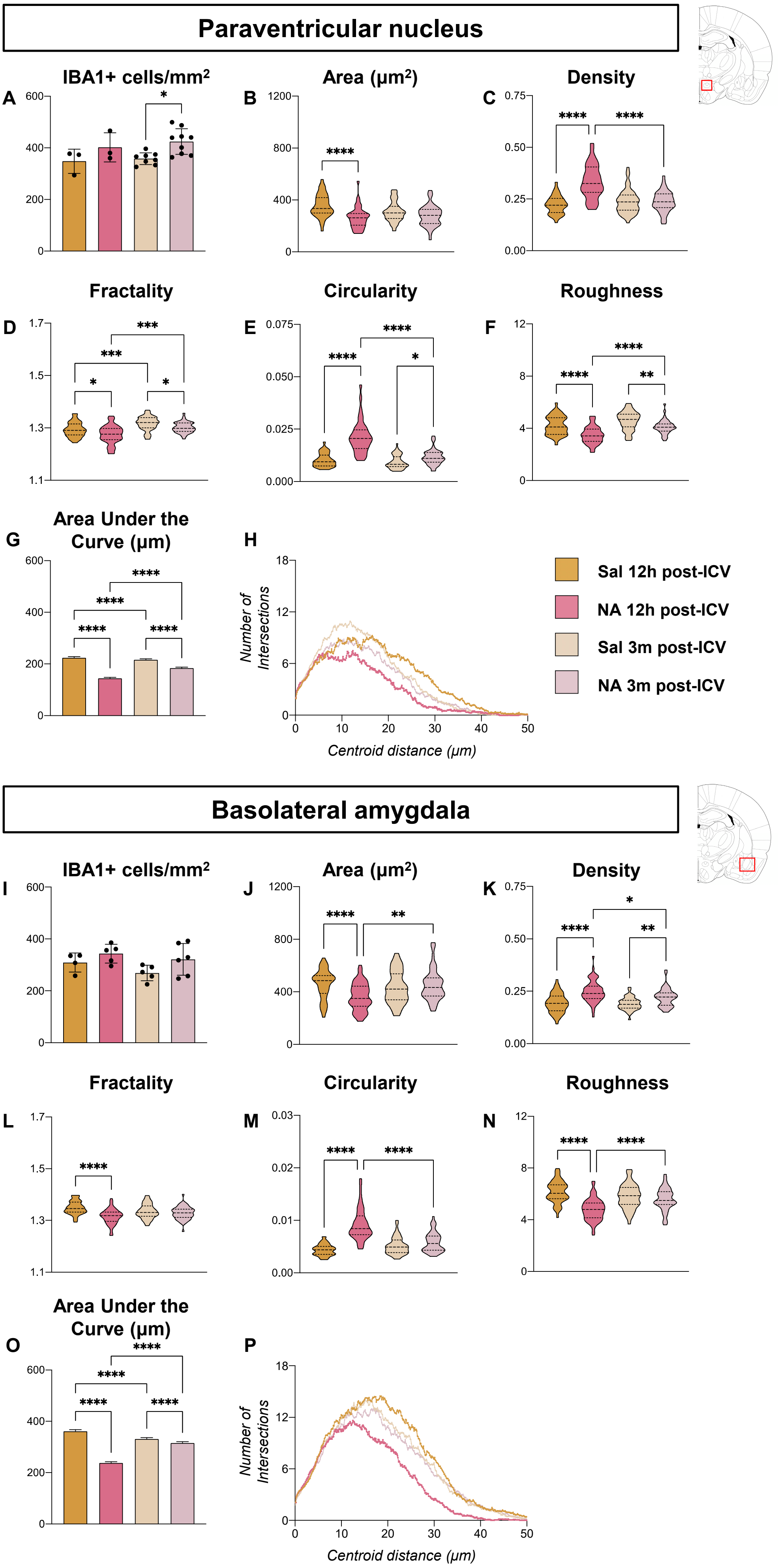

Paraventricular nucleus
A
IBA1+ cells/mm²
B
Area (µm²)
C
Density
D
Fractality
E
Circularity
F
Roughness
G
Area Under the Curve (µm)
H
Number of Intersections
Centroid distance (µm)
Sal 12h post-ICV
NA 12h post-ICV
Sal 3m post-ICV
NA 3m post-ICV
Basolateral amygdala
I
IBA1+ cells/mm²
J
Area (µm²)
K
Density
L
Fractality
M
Circularity
N
Roughness
O
Area Under the Curve (µm)
P
Number of Intersections
Centroid distance (µm)

Figure S3. Morphological analysis of microglial cells 12 hours and 3 months after ICV-injection of neuraminidase. Rats were intracerebroventricularly (ICV) injected with neuraminidase (NA) or saline (Sal) and sacrificed shortly after injection (12 hours) or 3 months later. Coronal sections at the level of the paraventricular nucleus (PVN) and the basolateral amygdala (BLA) were immunostained for IBA1 to label microglia. (**A**, **I**) IBA1-positive cell counts in the PVN (**A**) and the BLA (**I**); the histograms show the mean ± SD of n = 3-9 animals (for PVN) and n = 4-6 animals (for BLA) per group. (**B**-**F**, **J**-**N**) Morphological parameters obtained from Fractal analysis. Data distribution of each parameter is presented as violin plot, which has been truncated at the maximum and minimum values of each dataset; the dashed line represents the median and the dotted line the quartiles. (**G**, **H**, **O**, **P**) Data from morphological Sholl analysis. The *area under the curve* for the graphs of *number of intersections* over *distance from the centroid* (**H**, **P**) was calculated and presented as histograms (**G**, **O**), which show the mean ± SD of the n = 170-173 cells from PVN and n = 203-209 from BLA sampled from different animals within each experimental group. One-way ANOVA and Tukey's post-hoc were used as statistical tests. If the data met the assumption of normality and homoscedasticity, two-way analysis of variance (ANOVA) was employed, followed by Tuckey post hoc test for pairwise comparisons between groups. If the assumption of normality was not meet, Kruskal-Wallis test followed by Dunn's post hoc test for pairwise comparisons was used. If normality was assumed but not homoscedasticity, a Brown-Forsythe and Welch ANOVA test was employed, followed by Dunnett's T3 post hoc test for pairwise comparisons. In any of the cases, the groups were considered significant if $p < 0.05$. *$p < 0.05$, **$p < 0.01$, ***$p < 0.001$, ****$p < 0.0001$.
