# Supplementary material for: Persistent priming of hypothalamic microglia is associated with sensitization of the hypothalamic-pituitary-adrenal axis to acute stress, hyperactivity and behavioral response disruption in male rats
Source: Front Immunol. 2026 Jun 30;17:1828445. doi: 10.3389/fimmu.2026.1828445 (PMC13364640; doi:10.3389/fimmu.2026.1828445)
Supplement: Supplementary file 6 [file Image4.pdf]

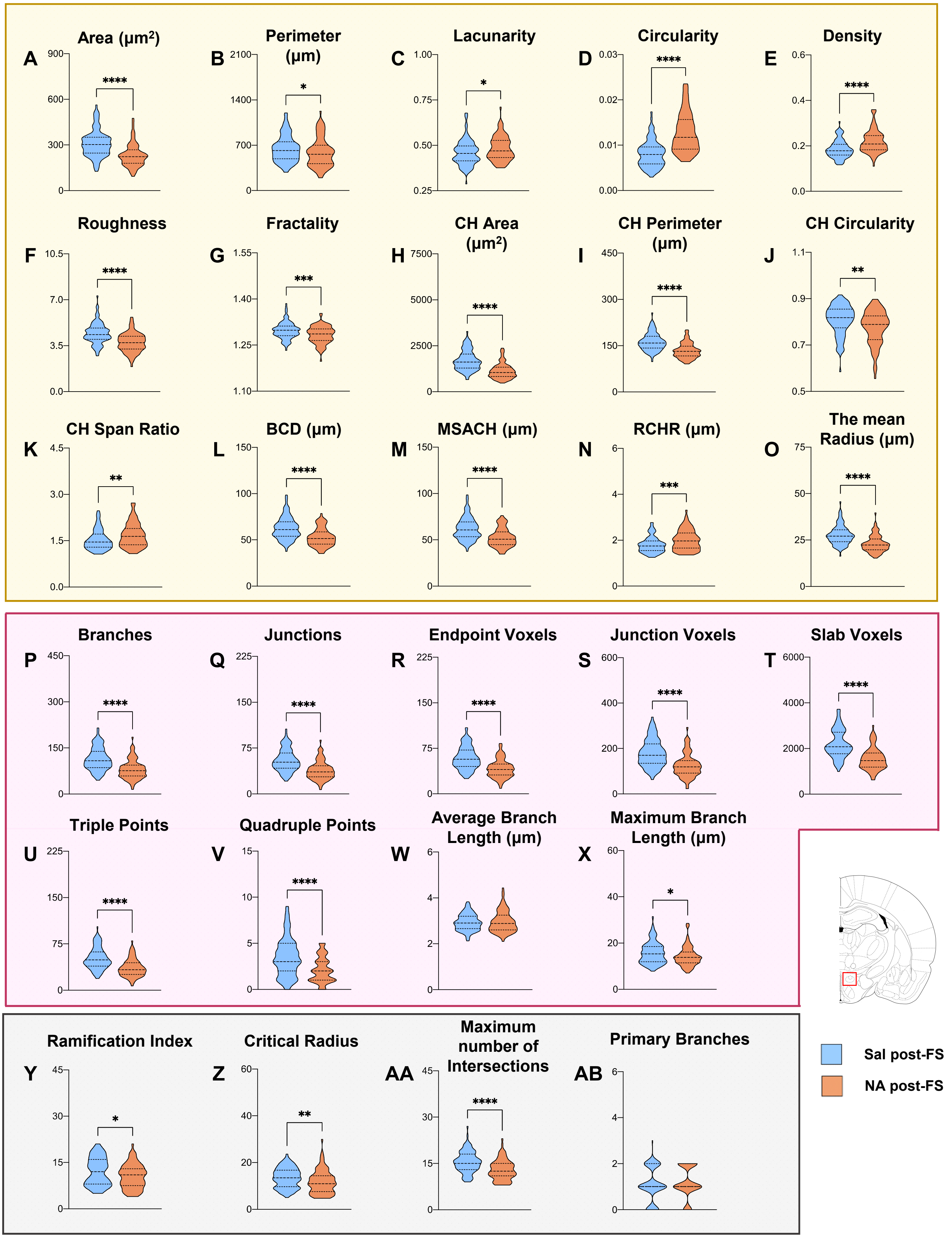
A
Area (μm²)
900
600
300
0
****
B
Perimeter (μm)
2100
1400
700
0
*
C
Lacunarity
1.00
0.75
0.50
0.25
*
D
Circularity
0.03
0.02
0.01
0.00
****
E
Density
0.6
0.4
0.2
0.0
****
F
Roughness
10.5
7.0
3.5
0.0
****
G
Fractality
1.55
1.40
1.25
1.10
***
H
CH Area (μm²)
7500
5000
2500
0
****
I
CH Perimeter (μm)
450
300
150
0
****
J
CH Circularity
1.1
0.9
0.7
0.5
**
K
CH Span Ratio
4.5
3.0
1.5
0.0
**
L
BCD (μm)
150
100
50
0
****
M
MSACH (μm)
150
100
50
0
****
N
RCHR (μm)
6
4
2
0
***
O
The mean Radius (μm)
75
50
25
0
****
P
Branches
450
300
150
0
****
Q
Junctions
225
150
75
0
****
R
Endpoint Voxels
225
150
75
0
****
S
Junction Voxels
600
400
200
0
****
T
Slab Voxels
6000
4000
2000
0
****
U
Triple Points
225
150
75
0
****
V
Quadruple Points
15
10
5
0
****
W
Average Branch Length (μm)
6
4
2
0
X
Maximum Branch Length (μm)
60
40
20
0
*
Y
Ramification Index
45
30
15
0
*
Z
Critical Radius
60
40
20
0
**
AA
Maximum number of Intersections
45
30
15
0
****
AB
Primary Branches
6
4
2
0
Sal post-FS
NA post-FS

Figure S4. Morphological analysis of IBA1 stained microglial cells sampled from the paraventricular nucleus of the hypothalamus. Rats were ICV-injected with neuraminidase or saline, and exposed to forced swimming 3 months later. They were euthanized 48 hours after FS. Morphological analysis carried out by three different methods: Fractal analysis (**A-O**, parameters enclosed in the yellow area), Skeleton analysis (**P-X**, parameters enclosed in the pink area) and Sholl analysis (**Y-AB**, parameters enclosed in the gray area). Data distribution of each parameter is presented as violin plot, where the dashed line represents the median and the dotted line represents the quartiles. N = 155-190 were sampled from different animals within each experimental group. *$p < 0.05$, **$p < 0.01$, ***$p < 0.001$, ****$p < 0.0001$.
