# Supplementary material for: Persistent priming of hypothalamic microglia is associated with sensitization of the hypothalamic-pituitary-adrenal axis to acute stress, hyperactivity and behavioral response disruption in male rats
Source: Front Immunol. 2026 Jun 30;17:1828445. doi: 10.3389/fimmu.2026.1828445 (PMC13364640; doi:10.3389/fimmu.2026.1828445)
Supplement: Supplementary file 7 [file Image5.pdf]

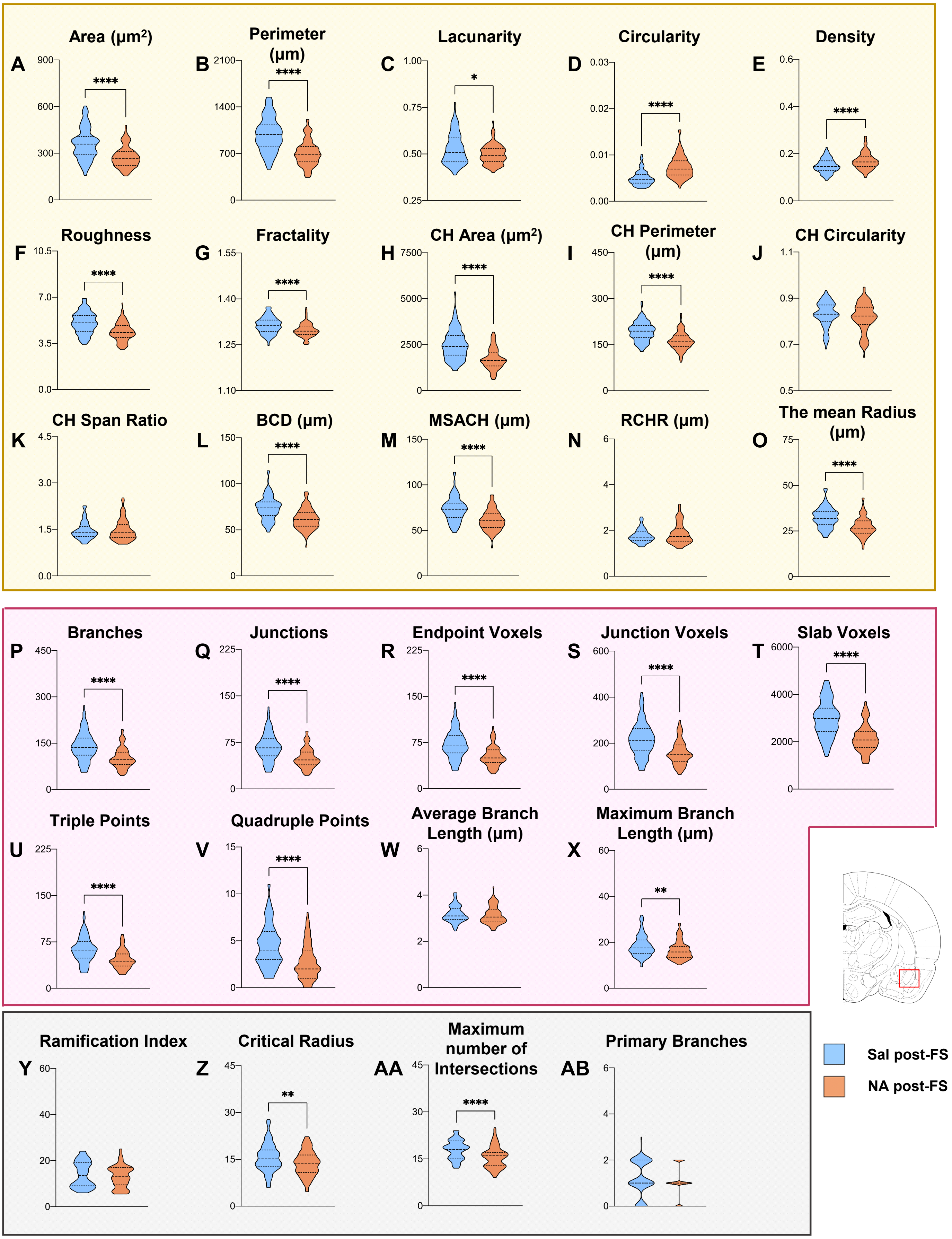

A Area (μm²) ****
B Perimeter (μm) ****
C Lacunarity *
D Circularity ****
E Density ****
F Roughness ****
G Fractality ****
H CH Area (μm²) ****
I CH Perimeter (μm) ****
J CH Circularity
K CH Span Ratio
L BCD (μm) ****
M MSACH (μm) ****
N RCHR (μm)
O The mean Radius (μm) ****
P Branches ****
Q Junctions ****
R Endpoint Voxels ****
S Junction Voxels ****
T Slab Voxels ****
U Triple Points ****
V Quadruple Points ****
W Average Branch Length (μm)
X Maximum Branch Length (μm) **
Y Ramification Index
Z Critical Radius **
AA Maximum number of Intersections ****
AB Primary Branches
Sal post-FS
NA post-FS

Figure S5. Morphological analysis of IBA1 stained microglial cells sampled from the basolateral amygdala. Rats were ICV-injected with neuraminidase or saline, and exposed to forced swimming 3 months later. They were euthanized 48 hours after FS. Morphological analysis carried out by three different methods: Fractal analysis (**A**-**O**, parameters enclosed in the yellow area), Skeleton analysis (**P**-**X**, parameters enclosed in the pink area) and Sholl analysis (**Y**-**AB**, parameters enclosed in the gray area). Data distribution of each parameter is presented as violin plot, where the dashed line represents the median and the dotted line represents the quartiles. N = 203-209 cells were sampled from different animals within each experimental group. *$p < 0.05$, **$p < 0.01$, ****$p < 0.0001$.
