# Supplementary material for: Persistent priming of hypothalamic microglia is associated with sensitization of the hypothalamic-pituitary-adrenal axis to acute stress, hyperactivity and behavioral response disruption in male rats
Source: Front Immunol. 2026 Jun 30;17:1828445. doi: 10.3389/fimmu.2026.1828445 (PMC13364640; doi:10.3389/fimmu.2026.1828445)
Supplement: Supplementary file 8 [file Image6.pdf]

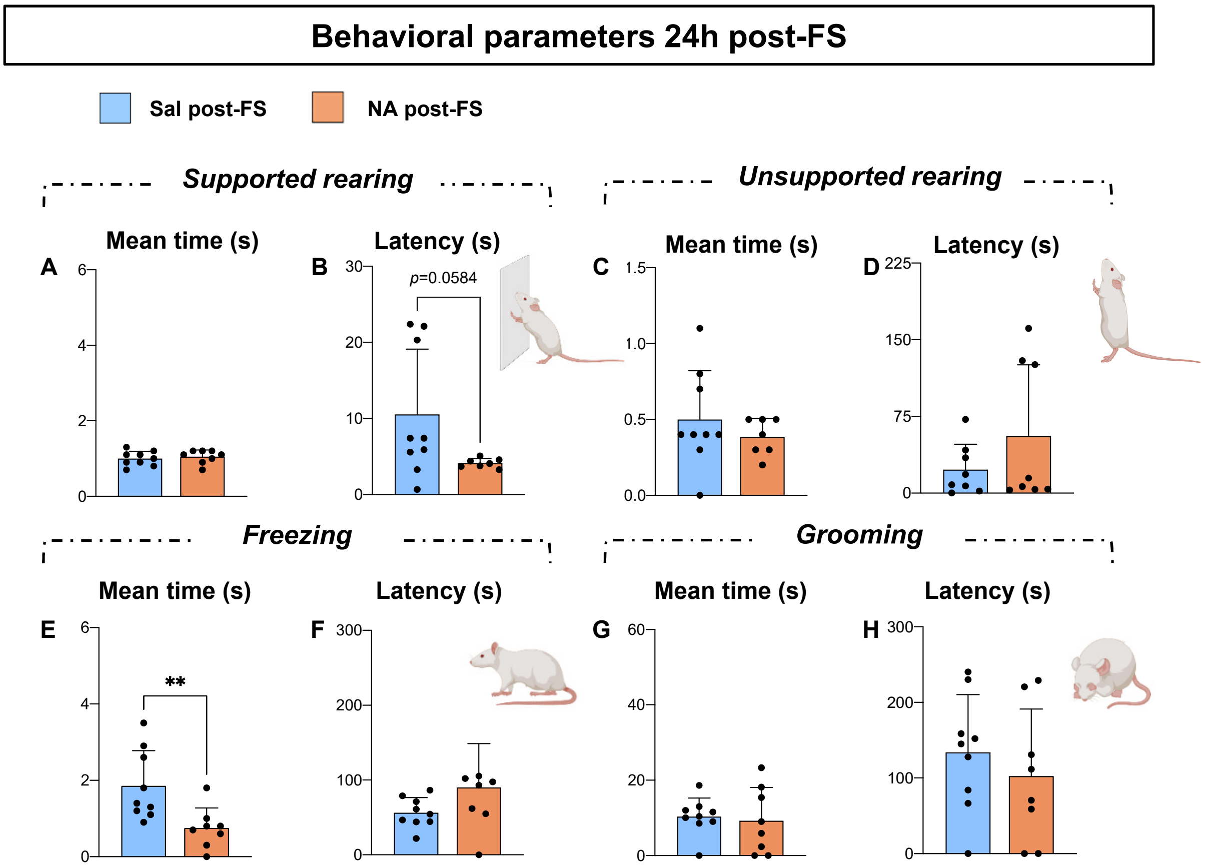

Figure S6. Parameters related to animal behavior in the open field. Rats were neuraminidase (NA) or saline (Sal) injected and, 3 months later, exposed to forced swimming (FS). After 24 hours, they performed an open field test. Parameters referring to supported rearing (**A-B**), unsupported rearing (**C-D**), freezing (**E-F**) and grooming (**G-H**) were measured. The histograms show the mean ± SD and the individual values of n = 8-9 animals per group. $^{**}p < 0.01$. p-values close to significance ($p < 0.15$) are also shown.
