# Supplementary material for: Persistent priming of hypothalamic microglia is associated with sensitization of the hypothalamic-pituitary-adrenal axis to acute stress, hyperactivity and behavioral response disruption in male rats
Source: Front Immunol. 2026 Jun 30;17:1828445. doi: 10.3389/fimmu.2026.1828445 (PMC13364640; doi:10.3389/fimmu.2026.1828445)
Supplement: Supplementary file 9 [file Image7.pdf]

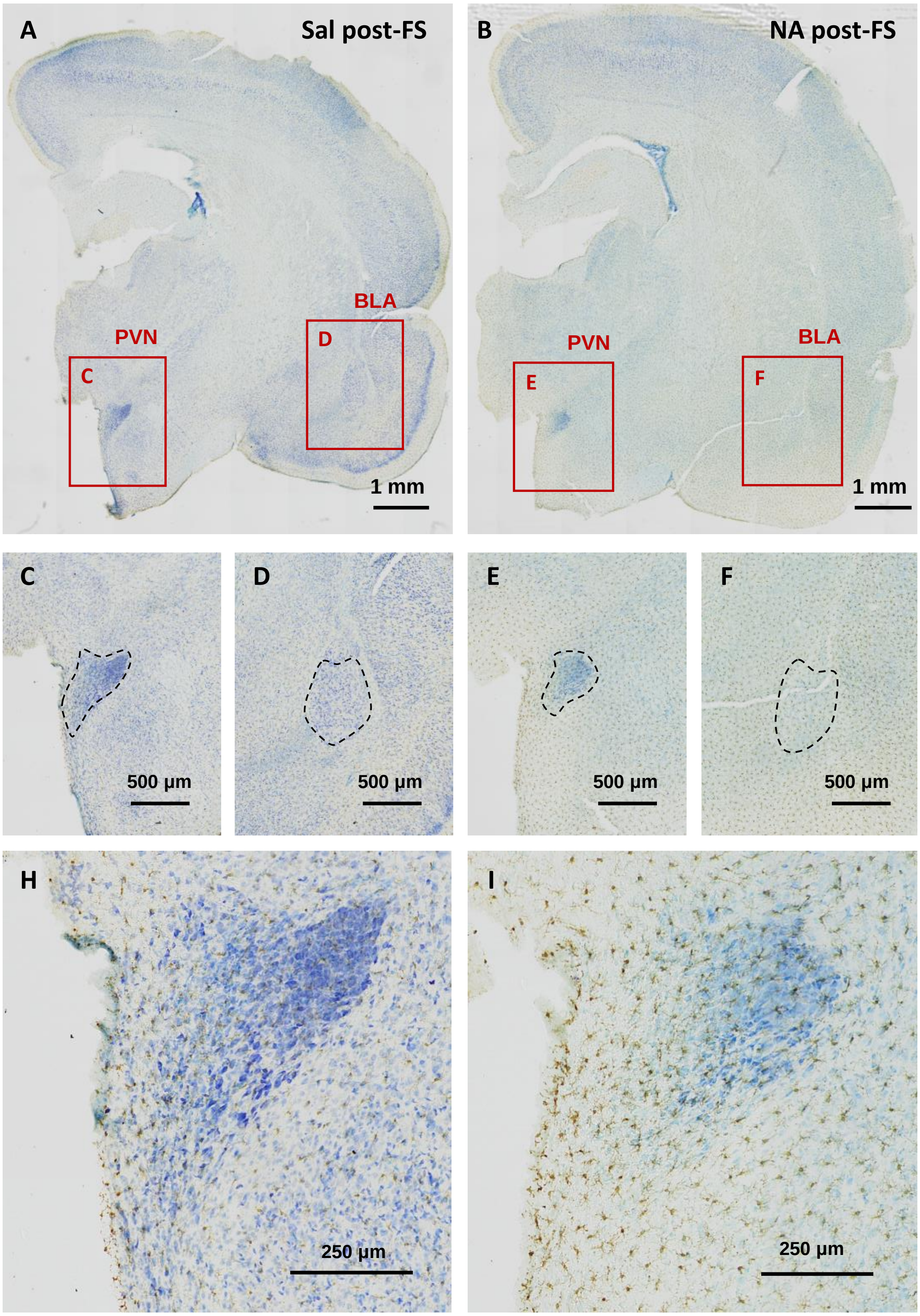

Figure S7. Microglia in the hypothalamic PVN and amygdala after NA-induced neuroinflammation and acute stress. Rats injected with neuraminidase (NA) or saline (Sal) were 3 months later exposed to forced swimming (FS). They were euthanized 48 hours after FS. Coronal brain sections at the level of the paraventricular nucleus (PVN) and the basolateral amygdala (BLA) were immunostained for IBA1 and counterstained with toluidine blue. (**A**, **B**) Low magnification images of representative sections from NA and saline injected rats, scanned using a 10x objective. (**C**, **E**, **H**, **I**) Higher magnifications of PVN. (**D**, **F**) Higher magnifications of BLA.
